# High pressure synthesis of phosphine from the elements and the discovery of the missing (PH_3_)_2_H_2_ tile

**DOI:** 10.1038/s41467-020-19745-2

**Published:** 2020-11-30

**Authors:** Matteo Ceppatelli, Demetrio Scelta, Manuel Serrano-Ruiz, Kamil Dziubek, Gaston Garbarino, Jeroen Jacobs, Mohamed Mezouar, Roberto Bini, Maurizio Peruzzini

**Affiliations:** 1 LENS, European Laboratory for Non-linear Spectroscopy, Via N. Carrara 1, I-50019 Firenze, Sesto Fiorentino Italy; 2 ICCOM-CNR, Institute of Chemistry of OrganoMetallic Compounds, National Research Council of Italy, Via Madonna del Piano 10, I-50019 Firenze, Sesto Fiorentino Italy; 3 ESRF, European Synchrotron Radiation Facility, 71 Avenue des Martyrs, 38000 Grenoble, France; 4 Dipartimento di Chimica “Ugo Schiff” dell’Università degli Studi di Firenze, Via della Lastruccia 3, I-50019 Firenze, Sesto Fiorentino Italy

**Keywords:** Chemical bonding, Solid-state chemistry, Physical chemistry

## Abstract

High pressure reactivity of phosphorus and hydrogen is relevant to fundamental chemistry, energy conversion and storage, and materials science. Here we report the synthesis of (PH_3_)_2_H_2_, a crystalline van der Waals (vdW) compound (*I*4*cm*) made of PH_3_ and H_2_ molecules, in a Diamond Anvil Cell by direct catalyst-free high pressure (1.2 GPa) and high temperature (T ≲ 1000 K) chemical reaction of black phosphorus and liquid hydrogen, followed by room T compression above 3.5 GPa. Group 15 elements were previously not known to form H_2_-containing vdW compounds of their molecular hydrides. The observation of (PH_3_)_2_H_2_, identified by synchrotron X-ray diffraction and vibrational spectroscopy (FTIR, Raman), therefore represents the discovery of a previously missing tile, specifically corresponding to P for pnictogens, in the ability of non-metallic elements to form such compounds. Significant chemical implications encompass reactivity of the elements under extreme conditions, with the observation of the P analogue of the Haber-Bosch reaction for N, fundamental bond theory, and predicted high pressure superconductivity in P-H systems.

## Abstract (web summary)

Chemical elements at high pressure may behave more consistently with their periodic properties than they do at ambient conditions. The authors report the synthesis of PH_3_ from black phosphorous and hydrogen, and the crystallization of the van der Waals compound (PH_3_)_2_H_2_ which fills a gap in the chemistry of adjacent elements in the periodic table.

## Introduction

The history of the layered structures of phosphorus has been intimately related to pressure since 1914^1^, when Percy Bridgman, who may be considered the founder of high pressure science, first synthesized black phosphorus (P_black_), whose characteristic crystalline layered structure corresponds to the thermodynamically stable allotrope of the element^2^. Rhombohedral A7, another layered structure of phosphorus, was later discovered by compression of P_black_ above 5 GPa^3^. Recently, a high pressure study, based on synchrotron X-ray diffraction (XRD) in Diamond Anvil Cell (DAC), has made another mark in the history of the layered structures of phosphorus, providing a clear insight about the mechanism of interlayer bond formation and significantly raising the pressure limit for the existence of the phosphorus layers up to ~30 GPa at room T^4,5^.

In 2014, the advent of phosphorene^6^, a 2D corrugated monoatomic layer of phosphorus atoms with extraordinary properties, whose stacking actually builds up the the crystal structure of P_black_, has sharply raised the attention of the scientific community about the layered structures of element 15, with considerable experimental and theoretical efforts nowadays in attempting to stabilize and functionalize the phosphorus layers by the introduction of molecular fragments^7^ or atoms, like for example H^8^.

During the last two decades, the generation of pressure in the GPa range has greatly expanded the horizon of chemistry under extreme conditions^9^. In particular, the combination of pressure with high temperature or electronic photo-excitation has been shown to be a very effective and extremely powerful tool for opening selective reactive paths^10,11^, activating chemical reactivity in notoriously non interacting systems at ambient conditions^12^, like here P and H, and for synthesizing new unexpected compounds^13,14^, thus suggesting the idea of investigating the so far unexplored chemistry of the phosphorus-hydrogen system under high pressure conditions.

Besides H-functionalization and stabilization of the layered structures of phosphorus, the direct chemical reactivity between elemental phosphorus and hydrogen under high pressure conditions is indeed currently of extreme interest for relevant issues essentially related to the chemistry and physics of phosphorus hydrides. Molecular hydrides of non-metallic elements have indeed always attracted the attention of high pressure chemists, physicists and materials scientists for their potential applications as superconducting^15^ and H-storage materials, due to their high H content and to the ability of forming stoichiometric van der Waals (vdW) compounds in the presence of H_2_^16,17^. Since the first report of a vdW solid made of He and N_2_^18^, many H_2_-containing vdW compounds involving elements from group 14 to group 18, including nobles gases, simple diatomics and molecular hydrides of non-metallic elements, have been experimentally observed at high pressure: CH_4_(H_2_)_4_^19^, CH_4_H_2_^19^, (CH_4_)_2_H_2_^19^, SiH_4_(H_2_)_2_^20^, GeH_4_(H_2_)_2_^21^, N_2_(H_2_)_2_^22^, (N_2_)_6_(H_2_)_7_^22,23^, (O_2_)_3_(H_2_)_4_^24^, (H_2_O)_6_H_2_^25^, (H_2_O)H_2_^25^, (H_2_S)_2_H_2_^26^, (H_2_Se)_2_H_2_^27^, (HI)_2_H_2_^28^, Ar(H_2_)_2_^29^, Kr(H_2_)_2_^30^, Xe(H_2_)_8_^31,32^. Nevertheless, among those formed by H_2_ and by the molecular hydride of a non-metallic element reported so far, none involves any of group 15 elements^33^. Within this picture, if the elements in the periodic table are to be considered tiles arranged on the basis of their electronic configuration, which determines their properties, then group 15 elements, named pnictogens, represent the missing tiles in this arrangement.

Phosphorus is here of particular relevance. Indeed the recent discovery of high superconducting *T*_c_ of 203 K in H_2_S at high pressure^34^, in agreement with the Bardeen-Cooper-Schrieffer (BCS) phonon mediated theory of high temperature superconductivity^35^, has further promoted the search for a similar behavior in the hydrides of neighbor elements in the periodic table. In particular, in the case of phosphorus the report of high pressure superconductivity in phosphine (*T*_c_ > 100 K, P > 200 GPa)^36^, has stimulated several theoretical studies aimed at exploring the structure and stability of PH_3_ at high pressure and the substantially unknown high pressure behavior of the phosphorus-hydrogen system, with the prediction of superconducting layered structures formed by these two elements above 80 GPa^37–39^.

Finally, the formation, stability and decomposition of PH_3_ in presence of H_2_ are relevant astrochemical issues^40^ related to the composition of giant planets, such as Jupiter and Saturn^41,42^, and their moons^43^, where PH_3_ and H_2_ have been detected.

**Fig. 1:**
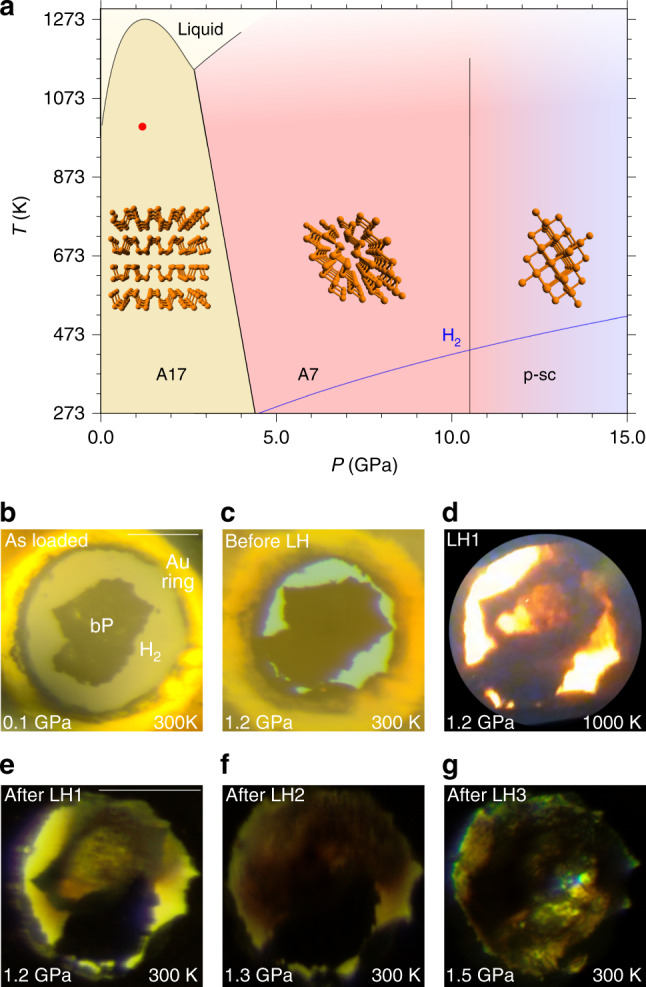
Phase diagram and reactivity of P and H_2_ at high pressure and high temperature. **a** Phase diagram of phosphorus (black lines), showing the stability regions of the orthorhombic (A17, P_black_), rhombohedral (A7) and pseudo simple-cubic (p-sc) structures^4,5^. The melting line of H_2_^73^ (blue line) and laser heating conditions (red point at *P* = 1.2 GPa, T ≈ 1000 K) are also displayed. **b**–**g** Microphotographs showing the sample aspect after loading (**b**), before, during and after LH1 (**c**–**e**), after LH2 (**f**), and after LH3 (**g**) at pressure ranging between 1.2 and 1.5 GPa (LH1, LH2, and LH3, respectively, indicate the first, the second and the third laser heating). The scale bars in the top right corner of **b**, **e** correspond to 100 μm.

Within this picture, in this paper we report a synchrotron XRD and vibrational spectroscopy (FTIR and Raman) study of the high pressure chemistry occurring between black phosphorus and molecular hydrogen at pressure of 1.2–1.5 GPa and temperature  ≲1000 K, where phosphorus is in the layered crystalline orthorhombic structure (A17), commonly known as black phosphorus, and H_2_ is liquid^44^ (Fig. 1). In these thermodynamic conditions PH_3_ is directly synthesized from the elements. On further room T compression, between 3.5 and 4.1 GPa, PH_3_ combined with excess H_2_ to form the crystalline vdW compound (PH_3_)_2_H_2_, whose identification has remarkable implications. Pressure was statically generated by means of membrane DAC and temperature by laser heating (LH), with P_black_ acting at the same time as reactant and laser absorber and H_2_ as a reactant and pressure transmitting medium.

## Results

### X-Ray Diffraction

After loading (see “Methods”), the sample of P_black_ and H_2_ was compressed to 1.2 GPa, where three laser irradiations were performed. The irradiations power (<3 W at laser output) and duration (up to  ~30 s) were carefully increased up to visually observe the real time chemical transformation of the sample, which occurred in the 900 ± 100 K temperature interval, with only a slight pressure drift from 1.2 to 1.3 GPa, and then to 1.5 GPa, respectively, after the second (LH2) and third (LH3) irradiation (Fig. 1).

**Fig. 2:**
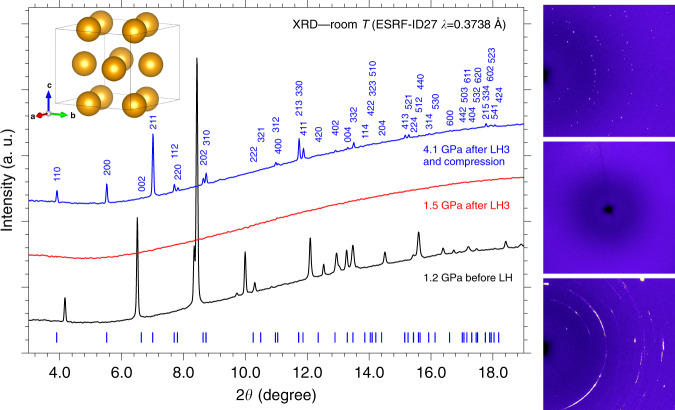
X-ray diffraction. Integrated panoramic XRD patterns and corresponding detector images of the sample, acquired at room T before laser heating (LH) at 1.2 GPa (black trace, bottom image), after the third laser heating (LH3) at 1.5 GPa (red trace, middle image), and after compression at 4.1 GPa (blue trace, top image). The blue ticks and labels respectively refer to the refined peak positions of the crystalline product at this pressure (Supplementary Note 1) and to the corresponding *h,k,l* indexing. The tetragonal *I*4*cm* structure of P atoms as obtained from single-crystal data at 5.5 GPa is also shown in the top left corner of the Figure (see the “X-Ray Diffraction” section).

Each irradiation produced an increasing transformation of P_black_, as confirmed by the intensity decrease of the P_black_ peaks in the diffraction patterns acquired before and after laser heating. After LH3 the absence of diffraction peaks indicated the complete consumption of P_black_, suggesting the formation of an amorphous or liquid product in the laser heated area (Fig. 2).

The sample was then compressed at room T and between 3.5 and 4.1 GPa the sudden appearance of single diffraction spots in the detector image, observed up to the highest explored pressure (5.5 GPa), unambiguously marked the formation of a crystalline product (Fig. 2). A careful XRD mapping of the sample over a grid with 10 μm spacing was performed to identify the regions where single spot diffraction could be observed. These regions were further explored by higher resolution mapping (4 μm spacing grid) and single-crystal patterns acquired on selected points of the laser heated area.

As H_2_ is liquid below 5.7 GPa (Fig. 1) and H atoms are weak X-ray scatterers, only information about the P atoms could be evinced from our single-crystal data, whose structure solution at 5.5 GPa indicates a tetragonal structure (*a* = 7.6075(6) Å, *b* = 7.6075(6) Å, *c* = 6.3303(13) Å, *α* = 90^∘^, *β* = 90^∘^, *γ* = 90^∘^, *V* = 366.36(9) Å^3^, *Z* = 8) belonging to space group *I*4*cm* (n. 108, ${C}_{{\rm{4v}}}^{10}$), where the shortest distance between P atoms is 3.608 Å (Fig. 2 and Supplementary Note 1) and which does not correspond to any known structure of phosphorus. An equivalent solution corresponding to centrosymmetric space group *I*4/*mcm* (n. 140, D${\,}_{4h}^{18}$) was initially considered (see “Discussion”).

The first reaction product that we thought about was of course PH_3_. Unfortunately, even if PH_3_ is expected to solidify at higher pressure with respect to NH_3_ (1.0 GPa) due to the smaller electric dipole moment and to the absence of H-bonding^45,46^, the solidification pressure of PH_3_ at room T is unknown.

Furthermore, the high pressure structure of solid PH_3_ is also unknown and only ambient pressure low T XRD data by Natta and Casazza dating back to 1930^47^ are available in the literature, indicating that PH_3_ crystallizes into a compact packing face centered cubic structure, possibly belonging to space group ${T}_{{\rm{h}}}^{2}$ (*Pn*$\bar{3}$, *P*2/*n*$\bar{3}$, n. 201) or ${O}_{{\rm{h}}}^{4}$ (*Pn*$\bar{3}$*m*, *P*4_2_/*n*$\bar{3}$ 2/*m*, n. 224), none of which is compatible with our single-crystal data (Supplementary Note 2).

Even considering the P positions in the *I*4*cm* and *I*4/*mcm* structures obtained from the single-crystal data as occupied by PH_3_ molecules, some inconsistencies emerge with a compact packing structure. Indeed, assuming orientationally disordered spherical shaped PH_3_ molecules in contact with each other, deriving the molecular volume using as molecular radius half of the shortest distance between P atoms ((4/3)*π*(3.608/2)^3^ = 24.592 Å^3^, in agreement with literature^48,49^), and considering 8 molecules per unit cell, then a filled volume of 196.736 Å^3^ out of the 366.36 Å^3^ unit cell volume obtained from the single-crystal data can be estimated, corresponding to a 0.537 filling ratio, which is significantly lower than the 0.74 ratio expected for a close packing structure.

This occurrence, indicating the presence of free volume, which can not be accounted for by a compact packing of PH_3_, provided the first hints suggesting the presence of interactions between PH_3_ molecules and a different composition of our reaction product, as indeed confirmed by the spectroscopic data.

### Fourier Transform InfraRed absorption spectroscopy

**Fig. 3:**
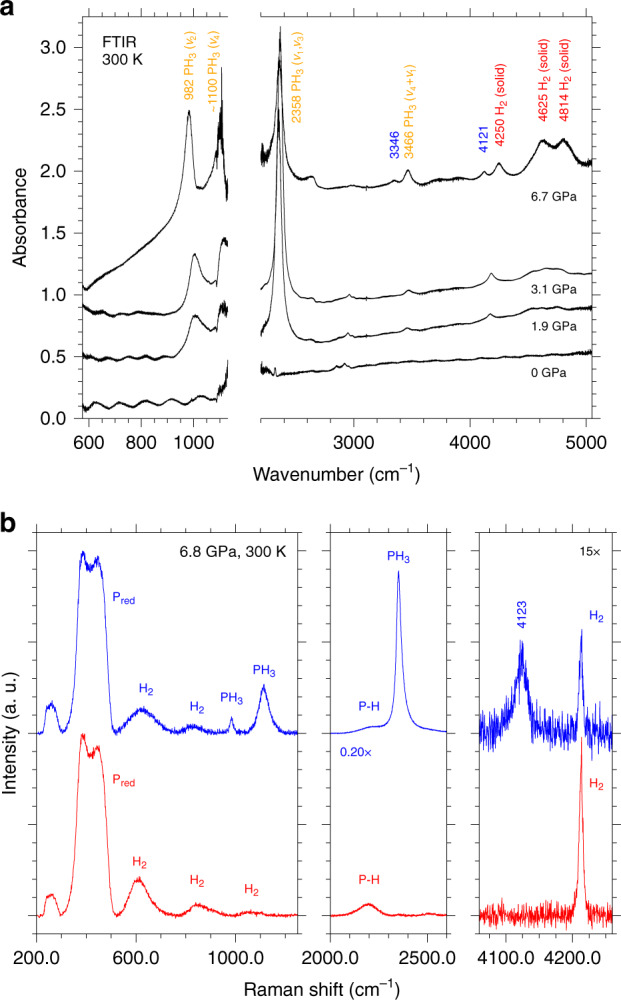
FTIR and Raman spectroscopy. **a** Room T FTIR absorption spectra of the sample acquired at 6.7 GPa after LH3 and at different pressure values during decompression to ambient conditions. The two bands at 3346 and 4121 cm^−1^ labeled in blue disappear on releasing pressure to 3.1 GPa. The spectra have been vertically translated for clarity and the values on the absorbance scale are intended for relative comparison. The break on the wavenumber axis excludes the spectral range corresponding to the Ia diamond saturating absorptions, which partially covers the *ν*_4_ absorption of PH_3_. The absorption features at  ~2640 cm^−1^ (6.7 GPa) and  ~2900 cm^−1^ (appearing on releasing pressure to 3.1 GPa) are assigned respectively to the diamond from the ambient pressure reference and to traces of oil on the optics of the interferometer. **b** Significant spectral regions of type-1 (red, lower trace) and type-2 (blue, upper trace) Raman spectra acquired on different selected spots of the mapping grid across the sample at 6.8 GPa and room T after LH. With respect to type-1 spectrum the type-2 one clearly shows the simultaneous presence of PH_3_ and of the extra band at 4123 cm^−1^ in the H_2_ stretching region at lower frequency compared to pure H_2_.

The Fourier Transform InfraRed (FTIR) spectra acquired after LH3 at 6.7 GPa and at different pressures during decompression are shown in Fig. 3 with the relevant band frequencies listed in Table 1. At 6.7 GPa, infrared absorption maxima are observed at 983,  ~1100 (out of scale) 2358, 3346, 3466, 4121, 4250, 4625, and 4814 cm^−1^.

**Table 1:** Infrared absorption frequencies at different pressure values.

IR bands	6.7 (GPa)	3.1 (GPa)	1.9 (GPa)	0 (GPa)	gas^52^	Liquid^52^	solid (35 K)^51^
PH_3 _*ν*_2_(*A*_1_) bend-s	983	1005	1002		992	~1100	980.3
PH_3 _*ν*_4_(*E*) bend-as	~1100^a^				1122	~1100	1095.4
PH_3 _*ν*_1_(*A*_1_) str-s, *ν*_3_(*E*) str-s	2358	2365	~2350^a^		2321 (*ν*_1_) 2327 (ν_3_)	2314	2303.6 (*ν*_1_) 2311.3 (*ν*_3_)
	3346						
PH_3_ (*ν*_1_ + *ν*_4_):(*ν*_3_ + *ν*_4_)	3466	3472	3458			3402	
	4121						
H_2_ (Q_1_(1))	4250	4184	4173				
H_2_ (Q_1_(1)+S_0_(0))	4625						
H_2_ (Q_1_(1)+S_0_(1))	4814						

Infrared absorption frequencies acquired at different pressure values during decompression to ambient pressure. The peak position of the *ν*_4_ band is not observable due to the overlap with the saturating absorption of the Ia diamond anvils. *ν*_1_ and *ν*_3_ are overlapped and unresolved. Literature values for PH_3_ are also reported.

*bend* bending, str stretching, *-s* symmetric, -as asymmetric.

^a^Out of scale absorption.

The last three bands can be assigned to infrared active absorptions of H_2_ (4250 (Q_1_(1), 4625 (Q_1_(1)+S_0_(0)), and 4814 (Q_1_(1)+S_0_(1)) cm^−1^) in agreement with literature^44^.

Even if the infrared bands of solid PH_3_ have been measured only at low T^50,51^ and those of liquid PH_3_ only at low temperature and modest high pressure (up to 35 atm)^52^, the bands observed at 983,  ~1100, 2358, and 3466 cm^−1^ can be confidently assigned to the fundamental and combination vibrational modes of PH_3_, as indicated in Table 1.

Finally, the two remaining bands at 3346 and 4121 cm^−1^ can not be assigned either to PH_3_ or H_2_ (Supplementary Note 3).

On decompression from 6.7 to 3.1 GPa, below the crystallization threshold of the reaction product, the two extra bands at 3346 cm^−1^ and 4121 cm^−1^ disappear. All the other bands of PH_3_ exhibit a high-frequency shift, as typically observed in H-bonded systems when releasing pressure, and the bands of H_2_ a low-frequency shift, as expected. On further decompression to ambient conditions, both PH_3_ and H_2_ bands shift to lower frequency, until disappearing with the opening of the cell.

No bands could be detected in the FTIR spectra after completely releasing the membrane pressure and opening/closing the cell under glove box in an inert atmosphere.

### Raman spectroscopy

A detailed Raman mapping, consisting of a 130 × 130 μm^2^ mesh with 10 μm grid spacing, was performed to gain insight about the reaction products and their spatial distribution within the sample at each pressure point during decompression, covering the entire frequency range between 40 and 4700 cm^−1^. The analysis of the Raman spectra acquired across the sample at 6.8 GPa revealed the presence of two limit spectra, referred to in the following respectively as type-1 and type-2 Raman spectra, and a variety of combinations of them, with significant bands in three spectral regions: 200–1250 cm^−1^, 2000–2600 cm^−1^, and 4000–4300 cm^−1^ (Fig. 3 and Supplementary Fig. 5).

Type-1 Raman spectrum (Fig. 3, lower panel, red trace) exhibits broad bands at 388, 449, 613, 833, 1064, 2198, 2520, and 4212 cm^−1^, whereas in type-2 Raman spectrum additional bands are detected at 984, 1114, 2352, 4123 cm^−1^. The two broad features observed in all the spectra at 388 and 449 cm^−1^ can be assigned to amorphous P_red_ (Supplementary Note 5). The weak band at 2198 cm^−1^, which has significantly lower frequency compared to phosphine (PH_3_ 2321 cm^−1^)^52^, or to higher phosphane homologues like diphosphane (P_2_H_4_ 2283 cm^−1^)^53^ and triphosphane (P_3_H_5_ 2267 cm^−1^ at 193 K)^53^, is compatible with the typical vibrational frequency of P–H bonds and further confirms the occurrence of chemical reactivity between P and H_2_. The sharp band at 4212 cm^−1^ identifies the characteristic stretching vibration of H_2_ and those at 613, 833, and 1064 cm^−1^ the corresponding pressure broadened S_0_(1), S_0_(2), and S_0_(3) rotational bands (S_0_(0) overlaps with the band of P_red_ at 388 cm^−1^)^54^.

Type-2 Raman spectrum exhibits additional bands at 984, 1114, and 2352 cm^−1^, which can be readily assigned respectively to the *ν*_2_, *ν*_4_ and *ν*_1_: *ν*_3_ fundamental modes of PH_3_^51^, consistently with the IR spectra (Fig. 3, lower panel, blue trace). However, differently from the IR spectra, no combination bands of PH_3_ are detected in the Raman spectra. The Raman band at 4123 cm^−1^, observed only in type-2 Raman spectra together with the presence of PH_3_, almost exactly matches the corresponding IR absorption band at 4121 cm^−1^ (6.7 GPa) and can not be assigned either to P, H_2_ or PH_3_.

As in the case of the IR spectra, during decompression to 3.1 GPa, below the crystallization pressure of the reaction product observed by XRD, the extra band at 4123 cm^−1^ disappears, while the bands of PH_3_ exhibit a high-frequency shift (Supplementary Fig. 5) and those of H_2_ a low-frequency shift. Both PH_3_ and H_2_ bands frequencies all undergo a low-frequency shift on further decompression and disappear after opening the cell.

At 1.95 GPa three sharp bands, unambiguously identified as the characteristic ${A}_{{\rm{g}}}^{(1)}$, *E*_g_ and ${A}_{{\rm{g}}}^{(2)}$ signatures of *P*_black_, respectively appear at 373, 443, and 467 cm^−1^, remaining observable on decompression down to ambient pressure (Supplementary Fig. 5). Even if the detection of these three peaks could suggest an incomplete transformation of *P*_black_ and its missed observation, the Raman spectra acquired at 0.2 GPa and at ambient pressure on the recovered sample show characteristic bands at  ~385 cm^−1^, which is not present in any of the *P*_red_ forms nor in *P*_black_ (Supplementary Fig. 7), and at 2245 cm^−1^ in the P–H stretching region, which closely resemble the Raman spectra reported by Yuan and coauthors for the recovered products of the decomposition of PH_3_ quenched from 25 GPa to 31 GPa^55^ (Supplementary Fig. 8), thus suggesting a laser-induced decomposition of PH_3_ during the acquisition of the Raman spectra, after releasing pressure below the crystallization threshold of the reaction product.

Interestingly, the P–H stretching band observed at 2200 cm^−1^ at 6.8 GPa exhibits a high-frequency shift to  ~2226 cm^−1^ on releasing pressure to 3.1 GPa, which further increases to 2245 cm^−1^ on releasing pressure to ambient conditions (Supplementary Fig. 5), providing evidence of the presence of H-bonding in the recovered solid product. Unfortunately, no additional insight could be gained on the recovered solid material responsible for type-1 Raman spectrum, which appears to consists of a hydrogenated (H-functionalized) mixture of amorphous *P*_red_ and crystalline *P*_black_.

## Discussion

Our data provide clear experimental evidence for direct high-pressure and high-temperature chemical reactivity between elemental P_black_ and H_2_. The resulting formation of PH_3_ according to the following chemical equation

1 $${\mathrm{P}}+\frac{3}{2}{{\mathrm{H}}}_{2}\,{{{1.2\, {\mathrm{GPa}}, 1000\,{\rm{K}}}\atop{-\!\!-\!\!-\!\!-\!\!-\!\!-\!\!-\!-\!\!-\!-\!\!\!-\!\longrightarrow}}\atop{{\rm{no}}\,{\mathrm{catalyst}}}}{\mathrm{PH}}_3$$

represents the so far unreported catalyst-free phosphorus analogue of the nitrogen-based Haber-Bosch reaction for the synthesis of NH_3_ (Supplementary Note 9).

Compressing PH_3_ in excess H_2_ at room T, between 3.5 and 4.1 GPa, the XRD data indicate the crystallization of a reaction product. As type-1 Raman spectrum was assigned to an amorphous solid product, we related the X-ray diffraction pattern of our crystalline product to type-2 Raman spectra, in which PH_3_ is observed, and considered the P positions of the corresponding structure to be occupied by PH_3_ molecules. The presence of PH_3_ in type-2 Raman spectra is always associated to the detection of an extra band in the H_2_ stretching region at lower frequency compared to pure H_2_, which disappears on releasing pressure below the crystallization threshold of the crystalline reaction product. A similar behavior is observed in the IR spectra, where an extra band is detected at 4121 cm^−1^ (6.7 GPa), almost exactly coinciding with the frequency of the extra Raman band at 4123 cm^−1^ (6.8 GPa).

Whereas the sharp higher frequency band perfectly matches with the literature data about H_2_ molecules in crystalline phase I (4250 cm^−1^ in IR and 4212 cm^−1^ in Raman at 6.8 GPa)^56^, the unassigned and unexpected broader extra band at lower frequency (4121 cm^−1^ at 6.7 GPa in IR and 4123 cm^−1^ at 6.8 GPa in Raman), indicates the presence of a second type of H_2_ molecules, which experience a significant weakening of the bond force constant (5.15%, average value between 4.30% Raman and 6.00% IR weakening), likely due to a different local force field, as indeed consistently attested by their larger bandwidth in comparison to pure H_2_.

**Fig. 4:**
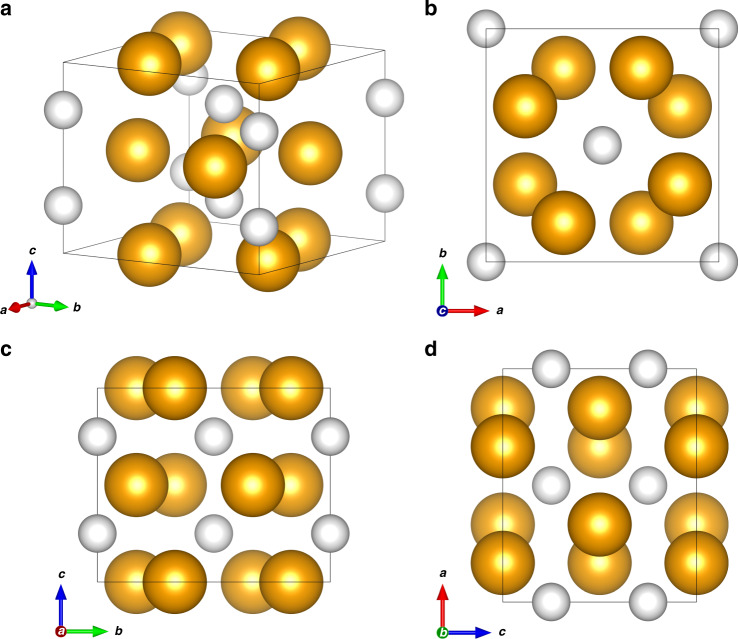
Crystal structure of the (PH_3_)_2_H_2_ vdW compound. Crystal structure of the (PH_3_)_2_H_2_ vdW compound at 5.5 GPa and room T (**a**) with views along the *c* (**b**), *a* (**c**), and *b* (**d**) crystal axes. The orange spheres represent the PH_3_ molecules, whereas the white spheres represent the H_2_ molecules. The size of the spheres has been drawn by estimating their radius as the sum of the P–H bond length (1.421 Å)^2^ and H van der Waals radius (1.20 Å)^49^ in the case of PH_3_ (2.62 Å) and as the sum of the H van der Waals radius (1.20 Å)^49^ and half of the H–H distance (0.742 Å)^74^ in the case of H_2_ (1.571 Å).

A possible interpretation for this occurrence is the formation of a van der Waals crystalline compound made of PH_3_ and H_2_ molecules with (PH_3_)_2_H_2_ stoichiometry and a tetragonal Al_2_Cu-like structure belonging to *I*4*cm* space group^26^, where PH_3_ and H_2_ respectively occupy 8*c* (*C*_*s*_) and 4*a* (*C*_4_) Wyckoff sites (Fig. 4). In this structure four molecules of PH_3_ are located on a plane parallel to the [a, b] direction at 0.5z and occupy the positions around a 4-fold rotation axis (*C*_4_) along the *c* direction. Four additional molecules occupy the positions generated by a rotation along *C*_4_ and a translation along +0.5z, giving rise to alternatively rotated layers of PH_3_ molecules.

This interpretation of our data is in agreement with the *I*4*cm* tetragonal structure of P atoms obtained from the single-crystal data and also accounts for the existence of free volume in the unit cell, in the case PH_3_ only would be present.

Furthermore, in this structure the H_2_ molecules are encaged within square antiprismatic voids delimited by eight PH_3_ molecules (four on one layer and four on the adjacent layer) and occupy a single type of crystal site (*C*_4_), corresponding to 4*a* Wyckoff positions, whose occupancy, according to group theory, is consistent with the appearance of one infrared and Raman active crystal component for the H_2_ stretching vibration (Supplementary Note 6 and Supplementary Fig. 10).

In addition, the occupation by PH_3_ molecules of 8*c* Wyckoff sites (*C*_*s*_), is consistent with the splitting of the (*ν*_1_ + *ν*_4_);(*ν*_3_ + *ν*_4_) combination band. The extra band observed in the IR spectra at 3346 cm^−1^ at 6.8 GPa, disappearing on decompression to 3.1 GPa, can be thus assigned to PH_3_ molecules forming the (PH_3_)_2_H_2_ crystal structure (Supplementary Note 6 and Supplementary Fig. 10).

A density of 1.269 g cm^−3^ can be calculated at 5.5 GPa from the refinement of the single-crystal data, with 2.89% in weight of H_2_ and total 11.5% in weight of H (H_2_ + H in PH_3_).

The molecular nature of the reaction product is further confirmed by the bulk modulus *B* = 6.7 ± 0.8 GPa derived from the 2nd order Birch-Murnaghan equation of state in the investigated pressure range, which is in absolute agreement with analogous systems^26^, and by the pressure evolution of the nearly constant *c*/*a* axial ratio, which indicates an almost isotropic compression within the applied pressure range (Supplementary Note 7).

Pressure has greatly extended the number of known hydrides synthesized under high-density conditions^15,33^. Among non metallic elements, H_2_-containing hydrides have been reported so far in literature for elements ranging from group 14 to group 18 of the periodic table, and include van der Waals hydrides of noble gases, simple diatomic molecules, and covalent molecular hydrides (Supplementary Fig. 16).

If among these hydrides we only consider those involving elements which are able to form covalent molecular hydrides (Supplementary Fig. 17) and particularly focus on those which have been reported to adopt a *I*4*cm* (*I*4/*mcm*) crystal structure with X_2_H_2_ composition, where X represents the corresponding molecular hydride (Supplementary Fig. 18), then we observe that this structure has been experimentally reported in the case of: carbon, with methane (CH_4_)^19^, for group 14; sulphur, with H_2_S^26^, and selenium, with H_2_Se^27^, for group 16; and iodine, with HI^57^, for group 17. Interestingly, to the best of our knowledge no such structure has been reported so far for any of the elements of group 15.

In particular (CH_4_)_2_H_2_, (H_2_S)_2_H_2_, (H_2_Se)_2_H_2_ and (HI)_2_H_2_ all reportedly exhibit the same *I*4/*mcm* structure, with the H_2_ and X molecules respectively occupying 4*a* and 8*h* Wyckoff positions, whereas (PH_3_)_2_H_2_ exhibits a *I*4*cm* structure with the H_2_ and PH_3_ molecules occupying the 4*a* and 8*c* Wyckoff positions. The *I*4*cm* and *I*4/*mcm* structures are closely related and only differ for the presence of an inversion center in *I*4/*mcm*, with identical lattice parameters and atomic positions. Noticeably, even if the occupation of 8*h* Wyckoff positions of *C*_2v_ site symmetry by CH_4_, H_2_S, H_2_Se and HI, respectively in (CH_4_)_2_H_2_, (H_2_S)_2_H_2_, (H_2_Se)_2_H_2_ and (HI)_2_H_2_ does not rise any symmetry issue (such as in the case of PH_3_ (*C*_3v_) occupying a *C*_2v_ site in the I4/*mcm* structure), no infrared spectra for any of these compounds have been acquired in the H_2_ stretching region, where, according to the analysis of the Davydov components activity using group theory arguments, the appearance of an extra band would unambiguously support the formation of a structure belonging to the *I*4*cm* rather than to the *I*4/*mcm* space group (Supplementary Note 6). Indeed, the possibility of the *I*4*cm* lower symmetry structure has been proposed also for (H_2_S)_2_H_2_^26^, whereas the *I*4/*mcm* structures of (H_2_Se)_2_H_2_ and (HI)_2_H_2_ were assigned according to similarity with (H_2_S)_2_H_2_, thus suggesting all these structures to belong to *I*4*cm* rather than *I*4/*mcm* space group. Furthermore, the consistency of the IR and Raman optical activity with the application of group theory to the crystal symmetry, indicates that the PH_3_ molecules are not randomly oriented and that their orientations reflect the symmetry and periodicity of the intermolecular potential originating from their symmetry. This apparently contrasts with the orientational disorder reported for CH_4_, H_2_S, H_2_Se, and HI, respectively, in (CH_4_)_2_H_2_, (H_2_S)_2_H_2_, (H_2_Se)_2_H_2,_ and (HI)_2_H_2_, which has been speculated from the behavior of the pure hydrides, without any conclusive evidence to support it like IR absorption spectra in the H_2_ stretching region (Supplementary Note 10)^58,59^. 

**Fig. 5:**
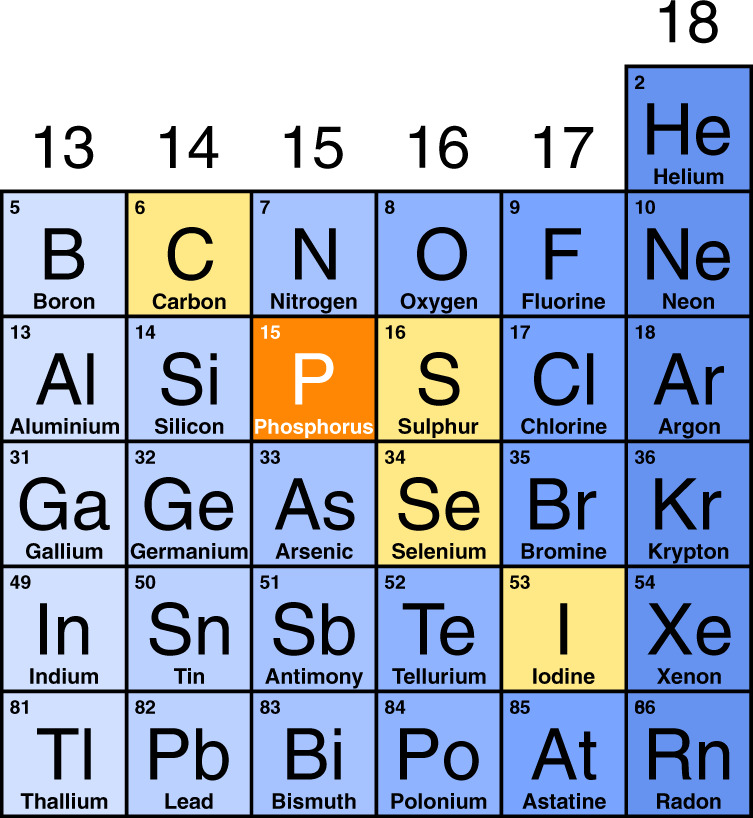
The missing tile of P for group 15. Non-metal region of the Periodic Table showing the elements (yellow and orange) able to form crystalline X_2_H_2_ vdW compounds (X = CH_4_^19^, PH_3_, H_2_S^26^, H_2_Se^27^, HI^57^) having the *I*4*cm* (*I*4/*mcm*) structure, with P (X = PH_3_), highlighted in orange, representing the so far missing tile for group 15 elements reported in this study (see text).

The identification of (PH_3_)_2_H_2_ thus represents the discovery of the missing tile for group 15, specifically corresponding to phosphorus, in the puzzle of the periodic properties of non-metallic elements, which are able to form van der Waals molecular compounds containing their covalent hydrides and H_2_ molecules (Fig. 5).

**Fig. 6:**
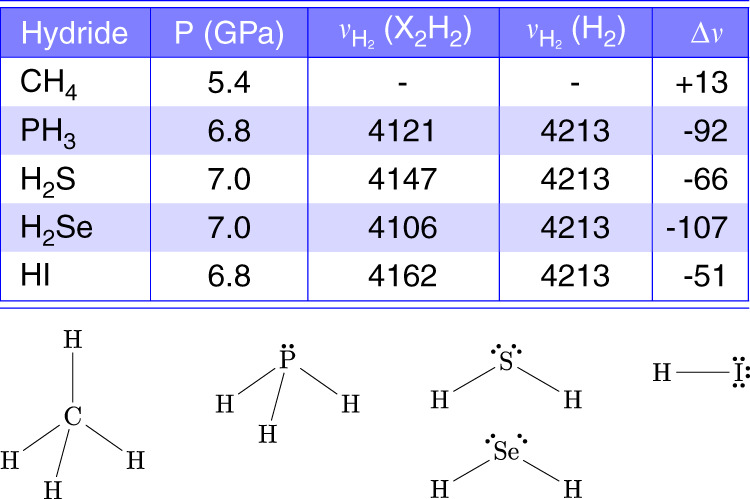
Raman shift of the H_2_ stretching vibration in different isostructural X_2_H_2_ compounds. Upper panel. Table reporting the Raman frequency (cm^−1^) of the H–H stretching vibration of H_2_ molecules in different isostructural X_2_H_2_ crystals (${\nu }_{{H}_{2}}$(X_2_H_2_) with X = CH_4_^19^, PH_3_, H_2_S^26^, H_2_Se^27^, and HI^57^) and their frequency shift (*Δν*) with respect to bulk H_2_ (${\nu }_{{H}_{2}}$(H_2_)) in comparable pressure conditions. Ref. ^19^ directly provides the frequency shift value for CH_4_. Lower panel. The molecular structures of CH_4_, PH_3_, H_2_S, H_2_Se, and HI, drawn according to the VSEPR theory and fulfilling the octet rule for the electron outer shell, are shown to highlight the absence of lone pairs on C in CH_4_, comparing to the other hydride forming elements.

A further chemical insight can be gained from the Raman data. All these five X_2_H_2_ isostructural compounds feature the extra Raman band in the H_2_ stretching region, due to the vibration of the H_2_ molecules inside their structure, in addition to the signal of the surrounding pure H_2_, which is always present as excess reactant from the synthesis. These frequencies are listed in the Table in Fig. 6, together with the corresponding frequency shift with respect to pure H_2_. An interesting feature emerging from this comparison is that, at similar high-pressure conditions, the frequency shift of the extra band with respect to pure H_2_ is always negative, except in the case of methane. According to the valence shell electron pair repulsion (VSEPR) theory, and to the fulfillment of the octet rule for the outer electronic shell^45^, the main difference between methane and the other hydrides is that methane does not possess an electron lone pair on carbon, whereas PH_3_, H_2_S, H_2_Se and HI all have at least one electron lone pair on the hydride forming element (Fig. 6).

The presence of lone pairs is typically associated to the ability of forming H-bonding, as indeed observed for all these systems, and of behaving as an electron donor Lewis base. H-bonding between the corresponding hydride molecules, evidenced by a negative frequency shift with increasing pressure of the internal stretching modes involving H atoms, has been indeed reported for (H_2_S)_2_H_2_, (H_2_Se)_2_H_2_, and (HI)_2_H_2_.

The high-frequency shift, observed both in the infrared and Raman spectra acquired on releasing pressure from 6.8 to 3.1 GPa across the melting threshold of the crystalline product (Supplementary Fig. 6), suggests also PH_3_ to behave like the analogous X_2_H_2_ isostructural van der Waals compounds, exhibiting H_3_P ⋯ H-PH_2_ H-bonding interactions, which disappear on decompression from 6.8 to 3.1 GPa after the decomposition of (PH_3_)_2_H_2_, as attested by the decrease of the vibrational frequencies of PH_3_ on further decompression.

The presence of a H-bonding between PH_3_ molecules has noticeable chemical relevance as PH_3_, in contrast to NH_3_, is known for not forming H-bonding at ambient conditions^45,46^, due to the small electronegativity difference of phosphorus with respect to hydrogen and to the consequent smaller electric dipole moment^60^. Furthermore, the existence of such interaction, together with the presence of H_2_ molecules, is in agreement with the larger volume of the crystalline cell of (PH_3_)_2_H_2_ compared to what expected in pure PH_3_.

The softening of the stretching vibration in the H_2_ molecules forming the (PH_3_)_2_H_2_ crystal clearly indicates the presence of chemical interaction between H_2_ and PH_3_. In the case of the isolated molecules such interaction has been described by ab initio computational methods^61^ in terms of two possible contributions: 1) the electron lone pair of P can act as a Lewis base and the σ^*^ anti-bonding molecular orbital of H_2_ as a Lewis acid (*n*  → σ^*^); 2) the *σ* bonding molecular orbital electrons of H_2_ act as Lewis base and the first anti-bonding molecular orbital of PH_3_ as a Lewis acid (*σ* → σ^*^(H-PH_2_)).

**Fig. 7:**
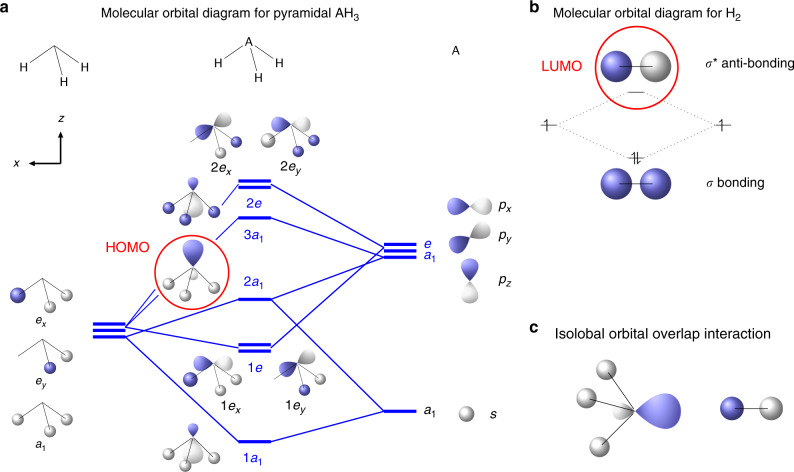
The molecular orbital interaction between PH_3_ and H_2_ in (PH_3_)_2_H_2_. **a** Qualitative molecular orbital diagram for pyramidal AH_3_ (where A = N, P, Sb, Bi) obtained from the symmmetry adapted linear combination of atomic orbitals of H_3_ (*C*_3v_) and A fragments (adapted from Figures 4–9 in ref. ^75^). The orbitals of each fragment are aligned vertically, in order of increasing energy from bottom to top, under the corresponding column of panel a, with H_3_ on the left side, AH_3_ in the middle and A on the right side. The 2*a*_1_ highest occupied molecular orbital (HOMO), where the electron lone pair is located, is highlighted by a red circle. **b** Molecular orbital diagram for H_2_, showing the completely filled *σ* bonding molecular orbital and the empty σ* anti-bonding lowest unoccupied molecular orbital (LUMO) highlighted by a red circle. **c** Isolobal orbital overlap interaction between the 2*a*_1_ molecular orbital of AH_3_ and the σ* anti-bonding molecular orbital of H_2_.

The first interaction is essentially a HOMO-LUMO orbital overlap interaction involving the highest occupied molecular orbital (HOMO) of PH_3_ (2*a*_1_ symmetry), which hosts the electron lone pair and has a prevalent non-bonding character, and the unoccupied σ^*^ anti-bonding molecular orbital of H_2_, technically the lowest unoccupied molecular orbital (LUMO), whereas the second one corresponds to the opposite situation, where the HOMO *σ* bond electron density of H_2_ interacts with the LUMO orbital of PH_3_ (3*a*_1_) (Fig. 7). Energetically, the first interaction is larger when the 2*a*_1_ HOMO of PH_3_ and the σ^*^ of H_2_ have maximum overlap, with the electron lone pair and the molecular axis of H_2_ aligned, but is present, even to a smaller extent, also in other interaction configurations, whereas the second one requires the *σ* electron density of H_2_ to interact with the 3*a*_1_ LUMO of PH_3_ in a configuration where the electron lone pair is perpendicular to H_2_ molecular axis.

In solid state, as group 15 elements are concerned, the first kind of interaction has been recently reported to be responsible for the softening of the H_2_ stretching vibration in the As_4_O_6_ ⋅ 2H_2_ crystal by electron density transfer from the As electron lone pair to the σ^*^ anti-bonding orbital of H_2_^62^. Gúnka et al. achieved this result by adopting the ICOHP (integrated projected crystal orbital Hamilton population) and ICOOP (integrated projected crystal orbital overlap population) computational methods, which, based on the crystal orbital overlap population (COOP) approach originally developed by R. Hoffmann^63^, indeed relate the local molecular orbitals to the band structure of crystals through the projection decomposition of the electron density of states, allowing to gain insight about the frontier orbitals that control structure and reactivity in extended systems.

Accordingly, a qualitative interpretation for the softening of the stretching vibration of the H_2_ molecules in the (PH_3_)_2_H_2_ crystal, certainly deserving appropriate theoretical investigation for effective electronic band structure calculation, is here proposed in terms of isolobal frontier molecular orbital overlap interaction between the HOMO of PH_3_, hosting the electron lone pair, and the σ^*^ anti-bonding LUMO of H_2_ (Fig. 7). Even if the orientation of PH_3_ and H_2_ molecules is not known, considering that H_2_ is expected to undergo hindered rotations at this pressure, it is indeed likely that the 2*a*_1_ HOMO of some of the PH_3_ molecules building up the cavities, where the H_2_ molecules are hosted, and the σ^*^ anti-bonding orbital of H_2_ dynamically adopt the correct orientation for an effective overlap. The σ^*^ anti-bonding orbital of H_2_ is normally not occupied, which makes the H_2_ molecule stable. The electron density transfer from the lone pair of PH_3_ to the σ^*^ anti-bonding orbital of H_2_ may decrease the bonding electron density of the H_2_ molecule, thus causing a reduction of the force constant (~5.15%) and finally a frequency decrease of the H_2_ stretching mode, according to the harmonic oscillator frequency equation (*ν* = $\frac{1}{2\pi }\sqrt{\frac{k}{\mu }}$). The existence of the *σ*(H_2_)  → σ^*^(H-PH_2_) interaction may further contribute to this effect. As a result, the H_2_ molecules within the crystal structures of (PH_3_)_2_H_2_ exhibit a lower vibrational frequency compared to bulk solid H_2_.

Finally, the observation of (PH_3_)_2_H_2_ is also somehow relevant for superconductivity in PH_3_. PH_3_ has been experimentally reported to become metallic at 40 GPa and superconducting at 207 GPa with a T_c_ of 103 K, but with no structural characterization so far^36^. Since then quite a lot of theoretical efforts have been made to account for such observation. At the moment, theory and experiments seem to agree about the instability of pure PH_3_ at high pressure, whose decomposition proceeds through the release of H_2_. Experimentally, a couple of recent papers have reported the decomposition of PH_3_^55,64^, with the initial formation of diphosphane followed by decomposition into elemental phosphorus and H_2_. However, no further convincing characterization was proposed, suggesting that, like in the case of H_2_S, other species may be responsible for the superconductivity observed by Drozdov et al.^36^. Theoretically, besides predicting the decomposition of PH_3_, different studies have calculated the stabilization of PH_2_ phases above 80 GPa, which, from a stoichiometric point of view, is consistent with the release of H_2_^37,38^. Furthermore, a key role of molecular H_2_ in stabilizing the high-pressure superconducting phases of phosphorus hydrides has been recently proposed^39^. Interestingly, even if the pressure range is here much lower, our data show that PH_3_ and H_2_ form a crystalline vdW compound, in which molecular H_2_ is indeed involved, possibly stabilizing PH_3_, or other related species, even at higher pressure.

To summarize, the results of this study have multiple chemically relevant implications. First of all, using LH in DAC, we have successfully induced direct reactivity between P_black_ and H_2_ at 1.2 GPa and temperature lower than 1000 K, without the use of any catalyst or precursor. To our knowledge this is the first report about a direct chemical reaction between P_black_, the thermodynamically stable allotrope of P, and H_2_ at high pressure and high temperature to form PH_3_, somehow mimicking and representing the P analogue of the Haber-Bosch process for the synthesis of NH_3_ from N_2_ and H_2_.

Secondly, at room T and pressure between 3.5 and 4.1 GPa PH_3_ combines with excess H_2_ to form the crystalline (PH_3_)_2_H_2_ van der Waals compound, whose observation consistently fills a gap existing for pnictogens in the periodic properties of non-metallic elements able to form crystalline vdW compounds made of the corresponding hydride and of molecular hydrogen. The identification of (PH_3_)_2_H_2_ represents the so far missing tile of P in this puzzle and confirms a general trend in the formation of H_2_-containing vdW compounds with X_2_H_2_ stoichiometry (X = molecular hydride) and *I*4*cm* (*I*4/*mcm*) structure.

The formation of unexpected chemical compounds made of components apparently non-interacting at ambient conditions, such as P and H, is extremely important for their relevant implications, which include H_2_ storage, the chemistry occurring in extraterrestrial environments of giant planets such as Jupiter, Saturn, and their moons, where PH_3_ and H_2_ are present^40,41,43^, and the identification of astrochemical processes leading to the synthesis of phosphine, which is a critical issue for the detection of the presence of life in harsh extraterrestrial environments of rocky planets, as inferred by the recent observation of anomalous high levels of phosphine in the cloud decks of Venus atmosphere^65^.

Third, as advancement in fundamental bond theory is concerned, the relevant observation of H-bonding in PH_3_, which in contrast to NH_3_ is not reported to exist at ambient pressure, and the existence of a molecular orbital interaction between the electron lone pair in PH_3_ and the antibonding molecular orbital of H_2_, provide remarkable insights to understand the effects underlying the predicted stabilization of the P–H systems under high-pressure conditions.

Finally, the synthesis of (PH_3_)_2_H_2_ effectively provides confirmatory experimental evidence for the key role played by H_2_ molecules in stabilizing the PH system at high pressure, as suggested by recent calculations predicting the presence of H_2_ units in superconducting PH structures at high pressure.

Once again the history of phosphorus has intertwined with pressure, whose role in enhancing similarities and consistencies in the periodic properties of elements apparently exhibiting particular behavior at ambient conditions^5,66,67^ is here further highlighted.

## Methods

### Synthesis of P_black_

Pure crystalline P_black_ was synthesized from red phosphorus according to reference^68^. All the reactants used for the synthesis of P_black_ were purchased from Sigma-Aldrich with the following purity: red phosphorus (>99.99%), tin (>99.999%), gold (>99.99%), and SnI_4_ (99.999%). The purity of the synthesized P_black_ crystals was checked by X-ray powder diffraction, Raman spectroscopy, EDX analysis and ICP-MS measurements, the latter giving a purity of 99.999+%. The crystals of P_black_ were fragmented by means of a metallic tip for obtaining smaller 20–40 μm chips to be loaded into the DAC.

### Sample preparation and the experimental conditions

Pressure was generated by means of membrane Diamond Anvil Cells (DAC) equipped with Ia type standard cut 16-sided beveled anvils having 600 μm culets. Re gaskets 200 μm thick were indented to 80 μm thickness and laser-drilled to obtain a 300 μm sample chamber. Before using them for the sideways containment of the samples, a Au ring was applied to prevent unintended catalytic effect and H_2_ diffusion and reactivity with Re. For this purpose the 300 μm diameter gasket hole was filled with Au powder, compressed with ~20 bar of He in the membrane until the powder appeared reflective and then laser-drilled again to obtain a 250 μm diameter hole. A small crystal of P_black_ was placed in the sample chamber by means of a metallic tip and the remaining volume was filled with fluid H_2_ using standard gas-loading technique. Au and a ruby chip were used to measure the pressure, whereas the temperature was measured by the fit of the black body thermal radiation emission of the sample during laser heating. High temperature was generated by means of Nd:YAG laser source (*λ* = 1064 nm) focused on the P_black_ crystal (≈30 μm beam spot size diameter), which acted both as reactant and laser absorber, thus avoiding any other source of contamination. No evidence for the formation of Au^69^ or Re^70^ hydrides was observed.

### X-ray diffraction data acquisition and analysis

XRD experiments were carried out at the ESRF-ID27 beamline using a monochromatic synchrotron radiation beam (*λ* = 0.3738 Å) focused to  ~5 μm to select different areas of the heterogeneous sample. The diffracted radiation was revealed by a MAR CCD165 detector, located approximately at 187 mm from the sample. The setup was calibrated against a CeO_2_ powder standard and Dioptas software was used to integrate the 2D area images to 1D patterns.

A single-crystal data set was collected at 5.5 GPa. Diffraction intensities were acquired in an *ω*-oscillation scan mode over the range  ±30^∘^ with a frame width of 0.5^∘^ and an exposure time of 2 s per single frame. The instrument model was calibrated at the beginning of the beam time by performing a full data collection of an enstatite single-crystal placed in a dummy DAC. The diffraction images were then imported into a CrysAlisPro suite (Supplementary Note 1) and processed accordingly. After determining the unit cell the intensities were reduced applying corrections for Lorentz and polarization effects, and also a multiscan absorption correction at the final step. Reciprocal lattice layers were reconstructed using the unwarp procedure (Supplementary Fig. 1). Careful inspection of the unwarped images did not reveal any twinning, satellite reflections or diffuse scattering, in contrast to the crystal of (H_2_Se)_2_H_2_, where diffuse scattering streaks were observed^27^. The crystal structure was subsequently solved by direct methods and then refined on F^2^ by full-matrix least-squares procedures using the SHELXL package (Supplementary Note 1). In addition, at the same pressure and at two other pressure points (4.1 and 4.5 GPa), panoramic oscillation images (*ϕ* = ±5^∘^, acquisition time 30 s) of oligocrystalline conglomerate were recorded and used to perform Le Bail fits with JANA 2006 software after azimuthal integration (Supplementary Figs. 2–4).

### Spectroscopic data acquisition and analysis

The Raman spectra were acquired at LENS with 1.5 cm^−1^ spectral resolution using the 647.1 nm emission wavelengths of a Kr ion laser. The details of the Raman setup are described elsewhere^71^. Raman spectra were acquired performing a 14 × 14 mapping over a 10 μm spaced grid using a single 300 groove/mm grating, which allowed to cover the 200–3300 cm^−1^ frequency region with 4 cm^−1^ spectral resolution. The most significant spots of the mapping were further inspected at higher resolution (1.5 cm^−1^) with different grating configuration down to 23.5 cm^−1^ (triple grating subtractive configuration 900–900–1800 groove/mm) and up to 4700 cm^−1^ (single grating configuration 900 groove/mm). No photochemical effect was observed at the employed laser power (1.5 mW) for pressure higher than 1.9 GPa, whereas at this pressure the formation of P_black_ could indicate the decomposition of PH_3_^55^.

The FTIR spectra were acquired with 1 cm^−1^ spectral resolution, using a Bruker IFS-120HR interferometer, suitably modified for the acquisition of infrared absorption spectra at high pressure in DAC^72^.

The frequency and intensity of the FTIR and Raman bands were obtained by fitting procedure using Voigt line shapes after baseline subtraction. Fityk software was used for this purpose.

## Supplementary information

Supplementary Information

## Data Availability

The X-ray crystallographic coordinates for the structure of the (PH_3_)_2_ vdW compound reported in this study have been deposited at the Cambridge Crystallographic Data Centre (CCDC), under deposition number 2034375. These data can be obtained free of charge from The Cambridge Crystallographic Data Centre via www.ccdc.cam.ac.uk/datarequest/cif. All other data that support the findings of this study are available from the corresponding author upon reasonable request.
