# Supplementary material for: High pressure synthesis of phosphine from the elements and the discovery of the missing (PH3)2H2 tile
Source: Nat Commun. 2020 Nov 30;11:6125. doi: 10.1038/s41467-020-19745-2 (PMC7705733; doi:10.1038/s41467-020-19745-2)
Supplement: Supplementary file 1 — Supplementary Information [file 41467_2020_19745_MOESM1_ESM.pdf]

Supplementary Information

High pressure synthesis of phosphine from the elements and the discovery of the missing $(PH_3)_2H_2$ tile

Matteo Ceppatelli,[*,†,‡] Demetrio Scelta,[†,‡] Manuel Serrano-Ruiz,[‡] Kamil Dziubek,[†,‡] Gaston Garbarino,[¶] Jeroen Jacobs,[¶] Mohamed Mezouar,[¶] Roberto Bini,[†,‡,§] and Maurizio Peruzzini[‡]

†*LENS, European Laboratory for Non-linear Spectroscopy, Via N. Carrara 1, I-50019 Sesto Fiorentino, Firenze, Italy*

‡*ICCOM-CNR, Institute of Chemistry of OrganoMetallic Compounds, National Research Council of Italy, Via Madonna del Piano 10, I-50019 Sesto Fiorentino, Firenze, Italy*

¶*ESRF, European Synchrotron Radiation Facility, 71 Avenue des Martyrs, 38000 Grenoble, France*

§*Dipartimento di Chimica "Ugo Schiff" dell'Università degli Studi di Firenze, Via della Lastruccia 3, I-50019 Sesto Fiorentino, Firenze, Italy*

E-mail: matteo.ceppatelli@iccom.cnr.it,ceppa@lens.unifi.it

Supplementary Note 1

X-Ray Diffraction (XRD). Dioptas software[1] was used to integrate the 2D area images to 1D patterns during the experiment. For the single crystal data set collected at 5.5 GPa the diffraction images were imported into a CrysAlisPro suite[2] and processed accordingly. The crystal structure was subsequently solved by direct methods and then refined on F^2 by full-matrix least-squares procedures using the SHELXL package.[3] The results are reported in the following Supplementary Tables 1, 2 and 3.

Supplementary Table 1: Experimental crystallographic refinement details for the $(PH_3)_2H_2$ van der Waals compound at room T and 5.5 GPa.

Crystal system	Tetragonal
Space group	$I4cm$
Space group IT number	108
a (Å)	7.6075 (6)
b (Å)	7.6075 (6)
c (Å)	6.3303 (13)
V (Å^3)	366.36 (9)
d (g cm^{-3})	1.269
abs. coeff (mm^{-1})	0.451
θ range (degree)	3.576-11.689
Limiting h index	$-7 \leq h \leq 7$
Limiting k index	$-7 \leq k \leq 8$
Limiting l index	$-4 \leq l \leq 5$
No. of measured reflections	175
No. of independent reflections	89
No. of reflections with I > 2σ(I)	73
No. of parameters	4
R_{int}	0.054
R[F^2, all data]	0.068
R[$F^2 > 2\sigma(F^2)$]	0.058
wR(F^2)	0.158
Goodness of fit	1.24
Highest diff. peak (e/Å^3)	0.316
Deepest diff. hole (e/Å^3)	-0.334

Supplementary Table 2: Fractional atomic coordinates and isotropic displacement parameters determined from the experimental crystallographic refinement of the $(PH_3)_2H_2$ van der Waals compound at room T and 5.5 GPa. Due to the low X-ray scattering cross-section of hydrogen and severe restrictions on data completeness implied by the diamond anvil cell, PH_3 and H_2 sites were modeled as P and H atoms, respectively.

	x	y	z	U_{iso}
P	0.1677(3)	0.6677(3)	0	0.0650(18)
H	0	0	0.25	0.03(3)

Supplementary Table 3: First and second neighbor $PH_3 \cdots PH_3$, $PH_3 \cdots H_2$ and $H_2 \cdots H_2$ intermolecular distances determined from the experimental crystallographic refinement of the $(PH_3)_2H_2$ van der Waals compound at room T and 5.5 GPa (calculated between the corresponding centers of mass). Due to the low X-ray scattering cross-section of hydrogen and severe restrictions on data completeness implied by the diamond anvil cell, PH_3 and H_2 sites were modeled as P and H atoms, respectively.

Distance	Length (Å)	Symmetry code
$P \cdots P_i$	3.608(6)	i = -x, 1-y, z
$P \cdots P_{ii}$	3.627(3)	ii = 1-y, 1-x, -1/2+z
$P \cdots P_{iii}$	3.627(3)	iii = 1-y, 1-x, 1/2+z
$P \cdots H_{iv}$	3.2439(8)	iv = x, 1+y, z
$P \cdots H_{v}$	3.2439(8)	v = -x, 1+y, -1/2+z
$P \cdots H_{vi}$	3.2439(8)	vi = 1/2+x, 1/2+y, -1/2+z
$P \cdots H_{vii}$	3.2439(8)	vii = 1/2-x, 1/2+y, z
$P \cdots H_{viii}$	5.471(2)	$viii$ = x, y, z
$P \cdots H_{ix}$	5.471(2)	ix = -x, y, -1/2+z
$P \cdots H_{x}$	5.471(2)	x = -1/2+x, 1/2+y, -1/2+z
$P \cdots H_{xi}$	5.471(2)	xi = -1/2-x, 1/2+y, z
$H \cdots H_{xii}$	3.1652(6)	xii = -x, y, 1/2+z
$H \cdots H_{xiii}$	3.1652(6)	$xiii$ = -x, y, -1/2+z
$H \cdots H_{xiv}$	5.3793(4)	xiv = -1/2-x, -1/2+y, z
$H \cdots H_{xv}$	5.3793(4)	xv = -1/2-x, 1/2+y, z
$H \cdots H_{xvi}$	5.3793(4)	xvi = 1/2-x, -1/2+y, z
$H \cdots H_{xvii}$	5.3793(4)	$xvii$ = 1/2-x, 1/2+y, z

.

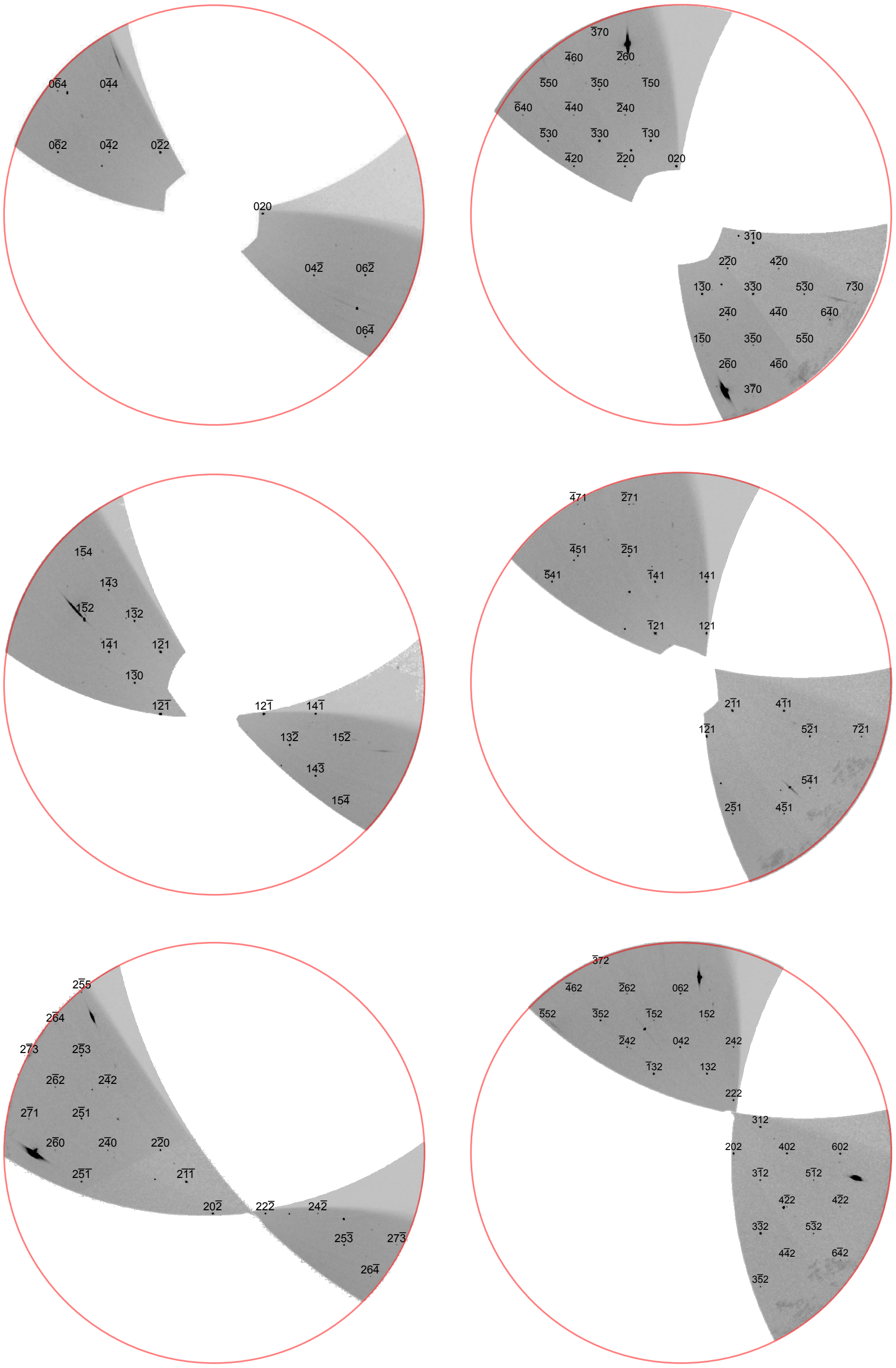

Supplementary Figure 1: The $0kl$, $1kl$, $2kl$ (left) and $hk0$, $hk1$, $hk2$ (right) layers of reciprocal space of $(PH_3)_2H_2$ reconstructed from diffraction images collected using a 2D CCD detector which demonstrate the tetragonal symmetry of the diffraction pattern. Note that the systematic absences are in a perfect agreement with the $I4cm$ space group symmetry. No diffuse scattering or satellite reflections are revealed. Additional spots and features placed out of the reciprocal lattice nodes can be attributed either to differently oriented crystal grains present in the part of the sample illuminated by X-rays or to the diamond anvils.

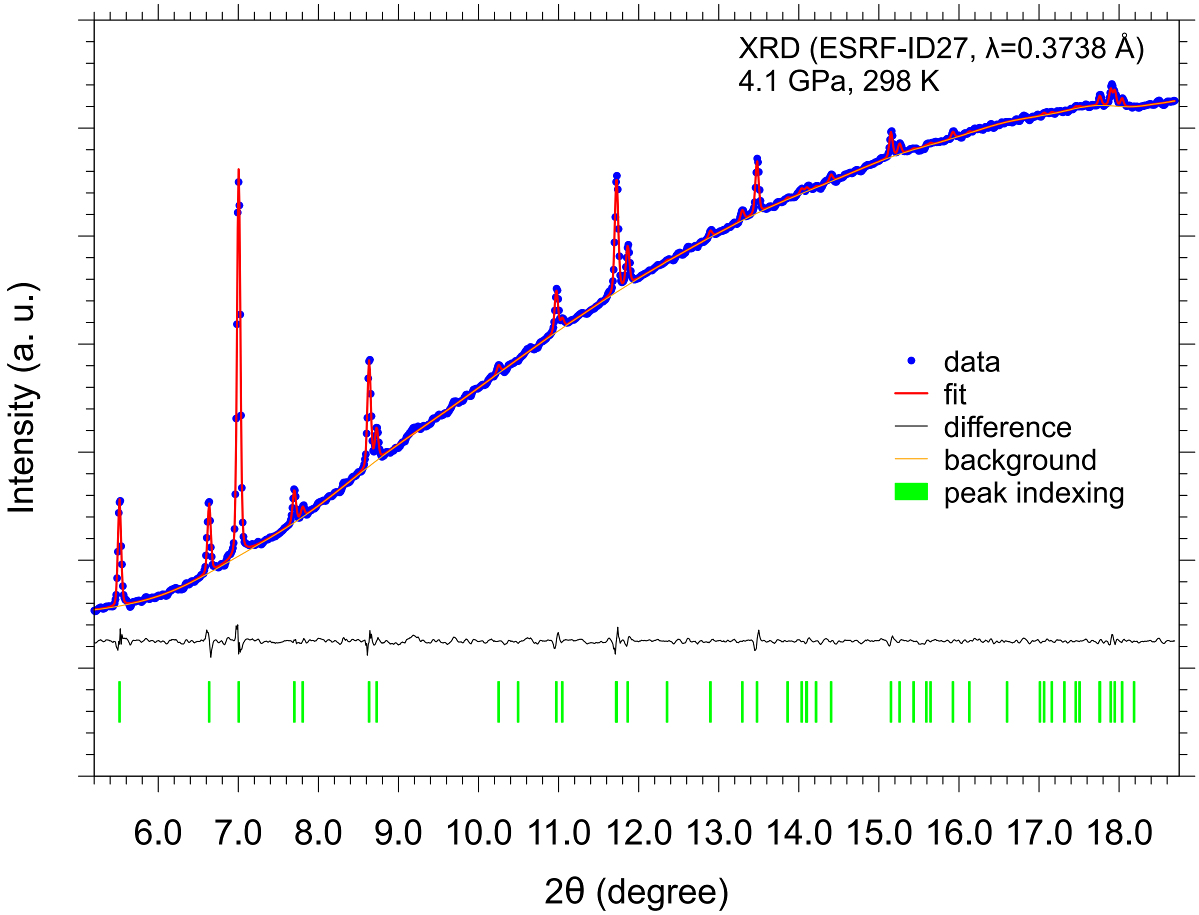

Supplementary Figure 2: Integrated panoramic XRD pattern (oscillation ϕ=±5°, acquisition time 30 s) and Le Bail crystal lattice refinement using JANA 2006 software[4] of the sample after laser heating at 1.2-1.5 GPa (T$\lesssim$1000 K) followed by room T compression at 4.1 GPa.

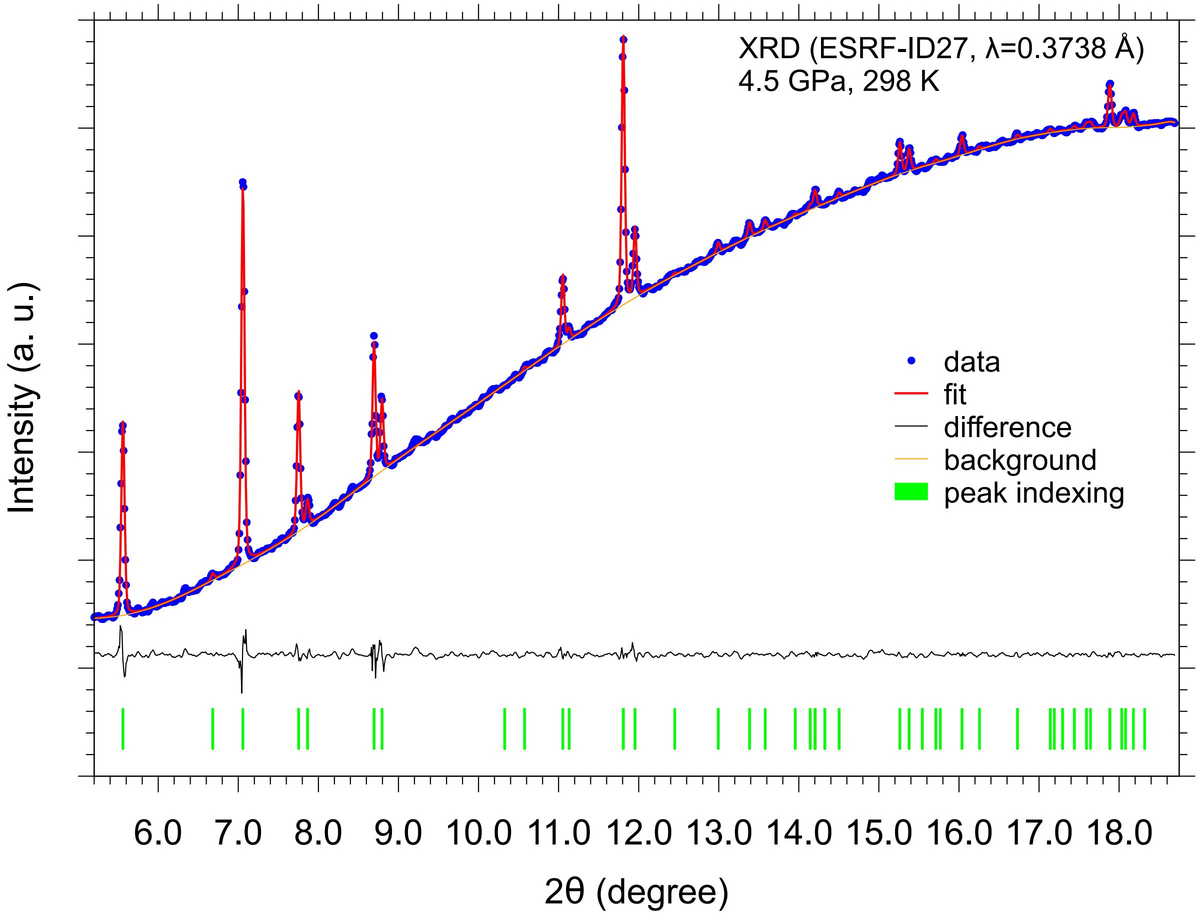

Supplementary Figure 3: Integrated panoramic XRD pattern (oscillation ϕ=±5°, acquisition time 30 s) and Le Bail crystal lattice refinement using JANA 2006 software[4] of the sample after laser heating at 1.2-1.5 GPa (T≲1000 K) followed by room T compression at 4.5 GPa.

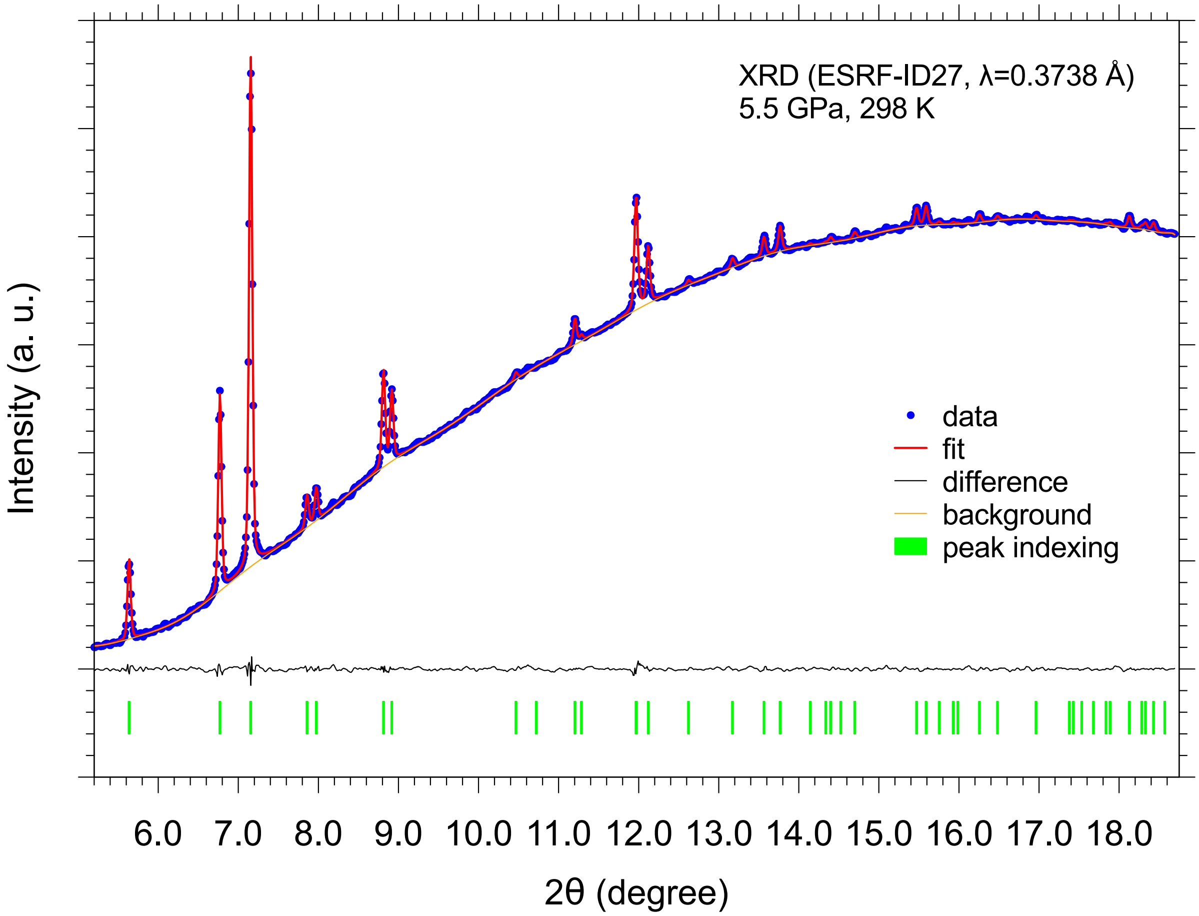

Supplementary Figure 4: Integrated panoramic XRD pattern (oscillation ϕ=$\pm$5°, acquisition time 30 s) and Le Bail crystal lattice refinement using JANA 2006 software[4] of the sample after laser heating at 1.2-1.5 GPa (T$\lesssim$1000 K) followed by room T compression at 5.5 GPa.

Supplementary Note 2

Phase diagram of PH_3. The solidification of PH_3 occurs at 140.65 K and the powder XRD data acquired at ∼-170 ℃ indicate that PH_3 crystallizes into a compact packing face centered cubic structure with four molecules in the unit cell (Z=4), possibly belonging to space group $T_h{}^2$ ($Pn\bar{3}$, $P2/n\bar{3}$, n. 201) or $O_h{}^4$ ($Pn\bar{3}m$, $P4_2/n\ \bar{3}\ 2/m$, n. 224) with lattice parameter a=6.31±0.01 Å, volume 251 Å^3 and density 0.896 g/cm^3, for which a filling ratio of 0.74 can be calculated.[5]

No high pressure structural data are available. PH_3 is expected to crystallize at higher pressure compared to NH_3 (1.0 GPa, 300 K)[6] due to the absence of H-bonding,[7–9] as expected from the different electric dipole moment ($\mu(NH_3)$=1.47 D, $\mu(PH_3)$=0.58 D)[10,11] and as indicated by the corresponding boiling temperature ($T_{boil}(NH_3)$=-33.34 °C, $T_{boil}(PH_3)$=-87.7 °C).

In our experiment, we did not observe any crystallization process on compression before 3.5-4.1 GPa, that could be ascribed to the formation of a PH_3 crystal, nor any phase separation.

These occurrences suggest that PH_3 formed after LH is liquid and miscible with excess H_2, forming a stable mixed liquid phase up to 3.5-4.1 GPa, where the $(PH_3)_2H_2$ vdW compound, which is evidently thermodynamically more stable than the PH_3 and H_2 components at these pressure and temperature conditions, crystallizes, similarly to what happens with the formation of the methane clathrate hydrate from liquid H_2O and CH_4.

Alternatively, PH_3 may give rise to an amorphous solid, which then reacts with liquid H_2 to form $(PH_3)_2H_2$, or to an amorphous solid mixture with H_2, which then crystallizes to form $(PH_3)_2H_2$. In both cases no Bragg peaks are expected in the diffractogram before the crystallization of $(PH_3)_2H_2$.

Supplementary Note 3

Fourier Transform InfraRed (FTIR) absorption spectroscopy. Considering the frequency difference (∼120 cm $^{-1}$) between the $\nu_2(A_1)$ and $\nu_4(E)$ bending modes, the band at 3346 cm^{-1} could be assigned to the $(\nu_1+\nu_2)$;$(\nu_3+\nu_2)$ combination mode, in a similar way as the band at 3466 cm^{-1} was assigned to $(\nu_1+\nu_4)$;$(\nu_3+\nu_4)$. Nevertheless the behavior of the 3346 cm^{-1} band on decompression proves this assignment to be incorrect. The frequency and intensity of the FTIR bands were obtained by fitting procedure using Voigt line shapes after baseline subtraction. Fityk software was used for this purpose.[12]

Supplementary Note 4

Raman spectroscopy. The frequency and intensity of the Raman bands were obtained by fitting procedure using Voigt line shapes after baseline subtraction. Fityk software was used for this purpose.[12]

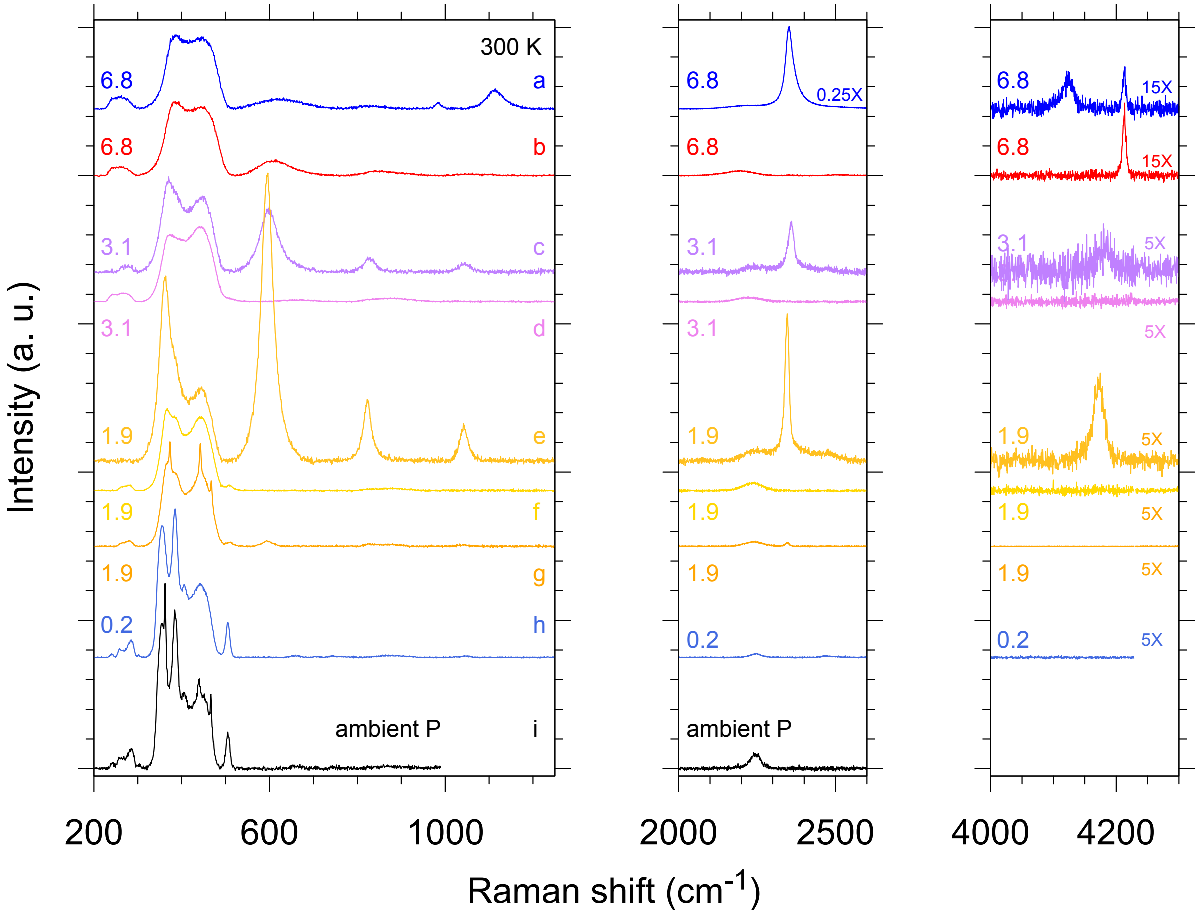

Supplementary Figure 5: Significant spectral regions of the Raman spectra acquired at 300 K after laser heating at different pressure points during decompression. Each spectrum was normalized to the band at ∼450 cm^{-1}. The labels of each spectrum indicate the corresponding pressure in GPa and the scaling factor for the intensity.

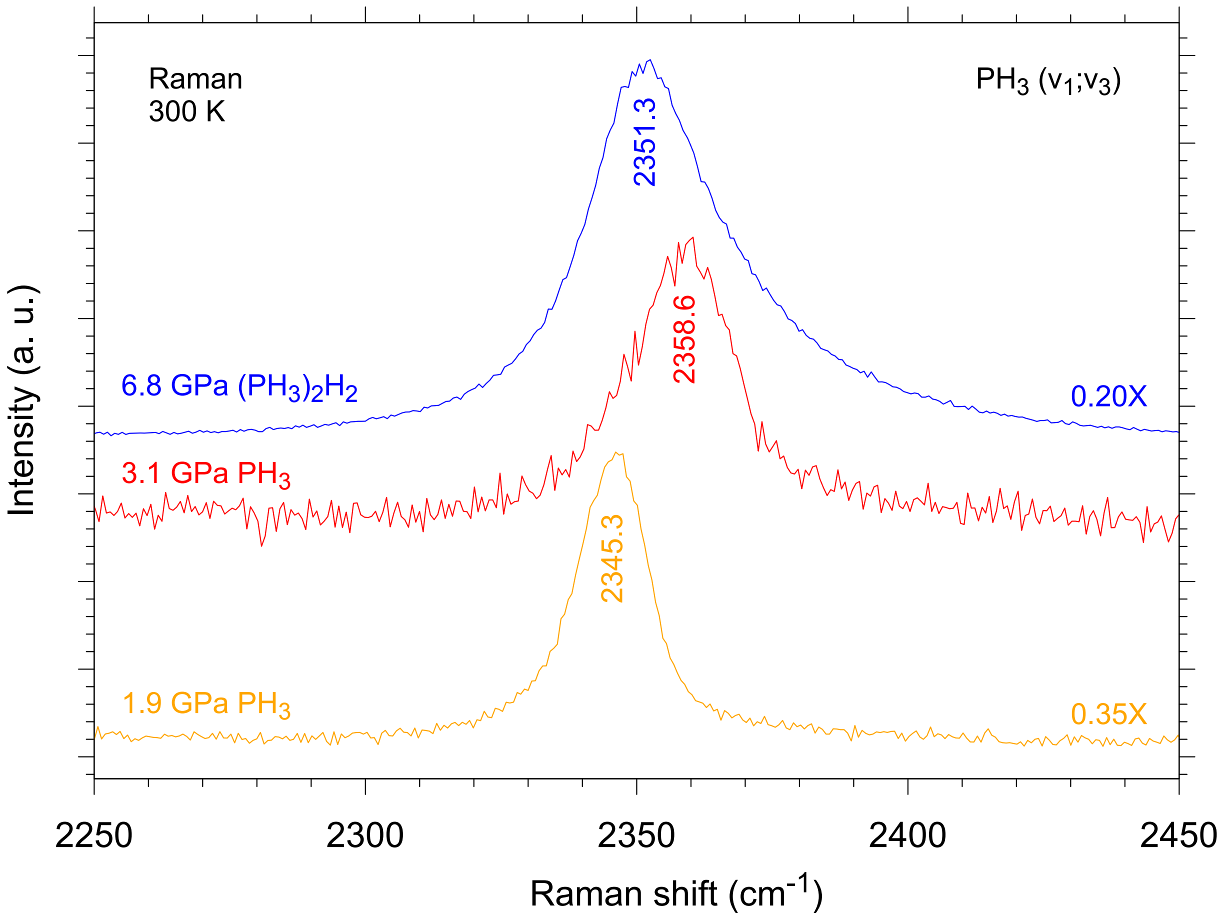

Supplementary Figure 6: Comparison among the Raman spectra of the sample in the ν_1;ν_3 stretching region of PH_3 at 6.8 GPa, 3.1 GPa and 1.9 GPa, illustrating the high frequency shift on releasing pressure from 6.8 GPa to 3.1 GPa across the melting pressure of $(PH_3)_2H_2$. The intensity of the spectra has been scaled, where indicated, and the spectra have been vertically translated for sake of clarity.

Supplementary Note 5

Product of type-1 Raman spectrum.

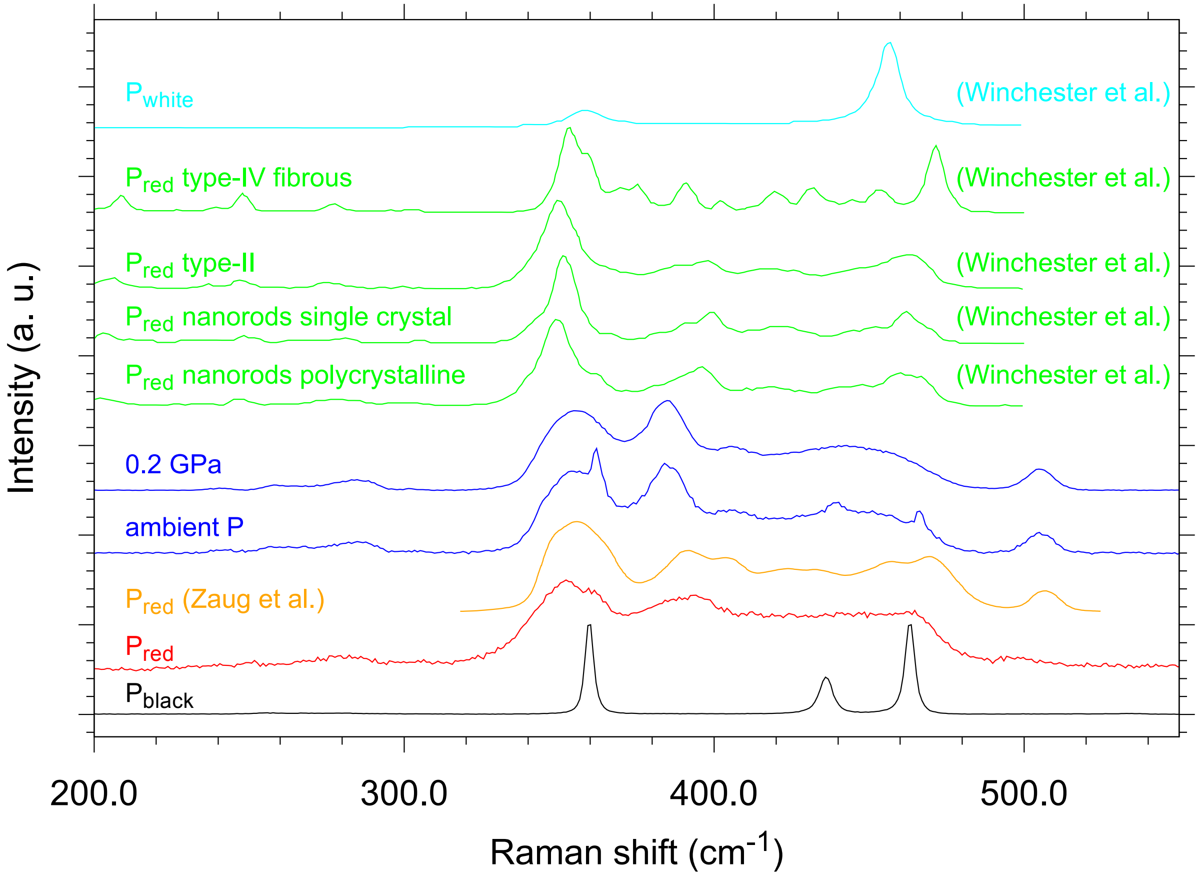

Supplementary Figure 7: Comparison among the Raman spectra of the recovered product at 0.2 GPa and at ambient conditions and those of P_{black}, P_{white} and different types of P_{red} from literature[13] at ambient conditions.

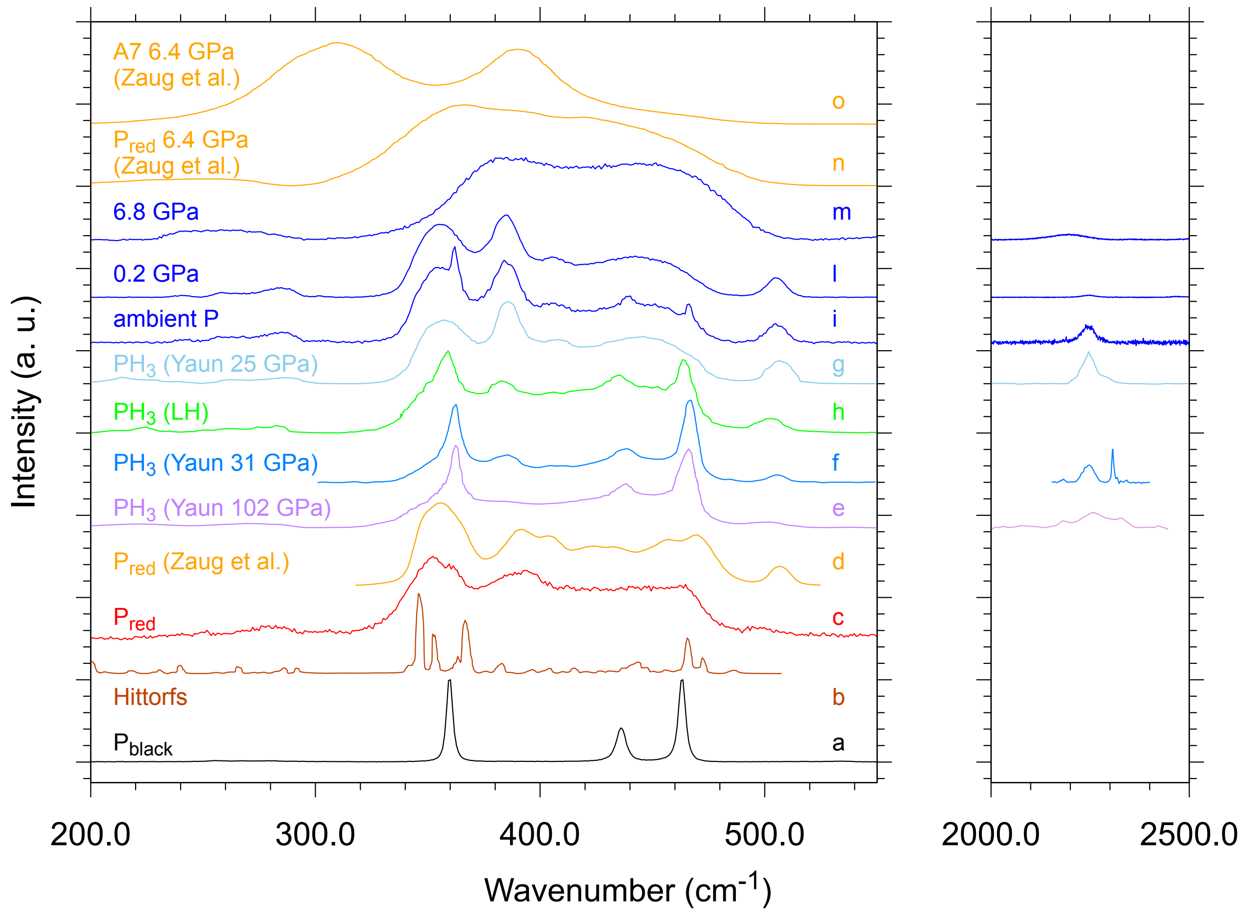

Supplementary Figure 8: Selected Raman spectral ranges showing a comparison among the Raman spectra of the sample acquired in this study at 6.8 GPa after laser heating (m), at 0.2 GPa after decompression (l) and at ambient pressure (i) and those of P_{black} (a, synthesized) and Hittorf's P (b, adapted from[14]) and amorphous P_{red} (c, from Aldrich) at ambient conditions. The Raman spectra of amorphous P_{red} from Zaug et al. at 6.4 GPa (n) and at ambient conditions (d) and of rhombohedral A7 phosphorus at 6.4 GPa (o) are also shown.[15] together with the spectra adapted from Yuan et al.[16] corresponding the recovered sample of PH_3, quenched from 25 GPa (g), 31 GPa (f), 102 GPa (e) and after laser heating (h).

Supplementary Note 6

Spectroscopic IR and Raman activity by group theory correlation diagrams. From the structure solution of the single crystal XRD data, without information on the position of the H atoms, the *I*4*cm* (C_{4v}^{10}) and *I*4/*mcm* (D_{4h}^{18}) structures are essentially indistinguishable. Nevertheless, according to group theory symmetry considerations about the optical activity of the IR and Raman bands, assuming the *I*4/*mcm* structure in the case of $(PH_3)_2H_2$ would imply the PH_3 molecules, having C_{3v} point symmetry, to inconsistently occupy *8h* Wyckoff positions of C_{2v} site symmetry. Moreover, no IR active component would results from the occupation by H_2 molecules of *4a* Wyckoff positions of D_4 symmetry (Supplementary Figure 9).

On the other hand, according to the International Tables for Crystallography Volume A, the space group 108 (*I*4*cm*, C_{4v}^{10}) features $C_4(4a)$, $C_{2v}(4b)$, $C_s(8c)$, and $C_1(16d)$ crystal sites, with the values in parentheses indicating the Wyckoff positions and the corresponding site multiplicity.[17,18] The structure solution of the XRD pattern of our crystalline product indicates a *I*4*cm* structure, in which the PH_3 molecules occupy the C_s (8c) sites, consistently with the molecular C_{3v} point symmetry (Supplementary Figure 10).

As the H_2 molecules are concerned, according to group theory, only the occupation of the C_4 (4a) sites gives rise to one IR and Raman active component in the Davydov crystal field splitting for the H-H stretching vibration (Supplementary Figure 10). The occupation of the other sites by H_2 molecules would indeed activate more components than the single extra band observed in the H_2 stretching region of both IR and Raman spectra and can thus be confidently excluded.

In particular, the occupation of the C_{2v} (4b) sites would produce one IR and Raman active component (A_1) and one Raman active component (B_1) (Supplementary Figure 11).

The occupation of C_s (8c) sites would produce two IR and Raman active (A_1 and E) components and one B_1 Raman active component (Supplementary Figure 12).

Finally, the occupation of the C_1 (16d) sites would produce three IR and Raman active

components (1 A_1, 2 E) and two Raman active components (1 B_1 and 1 B_2) (Supplementary Figure 13).

One may speculate and argue that the experimental observation of the single additional component expected for the occupation of the $C_{2\mathrm{v}}$ sites with respect ot the C_4 ones was somehow missed. Nevertheless, even if this would be the case, the occupation of $C_{2\mathrm{v}}$ rather than C_4 sites by H_2 molecules, would correspond to the same $(PH_3)_2H_2$ stoichiometry of the vdW compound, as the multiplicity of $C_{2\mathrm{v}}$ and C_4 sites is the same.

The crystal structure belonging to the $I4cm$ space group, where the PH_3 molecules occupy $8c$ Wyckoff positions of C_s site symmetry and the H_2 molecules occupy $4a$ Wyckoff positions of C_4 site symmetry, with a resulting $(PH_3)_2H_2$ stoichiometry determined by the full occupancy of the site multiplicity, fully and consistently accounts for all the issues related to the existence of free volume, in the case PH_3 would be present, and to the optical activity of the extra component appearing in the IR and Raman spectra of the crystalline vdW compound.

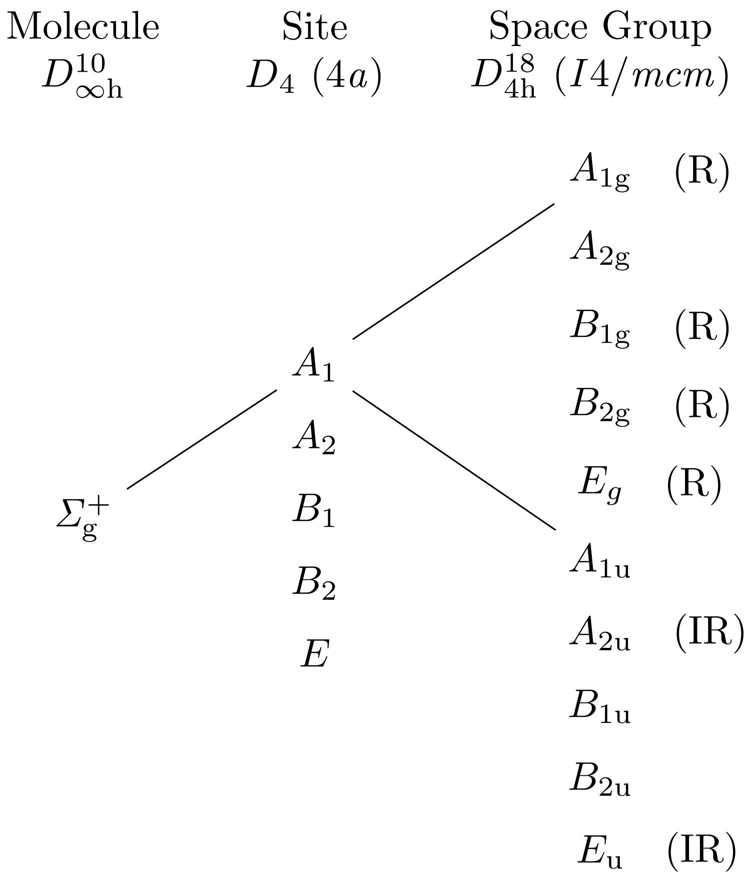

Supplementary Figure 9: Correlation diagram between the irreducible representations of the normal modes of vibrations of H_2 in the point symmetry group ($D_{\infty h}$) of the isolated molecule (left side) and in the $I4/mcm$ (D_{4h}^{18}) space group of the $(PH_3)_2H_2$ crystal structure (right side), through the corresponding site symmetry (center). The infrared (IR) and Raman (R) optical activity of the symmetry species are also reported. In this case only one Raman active component should be observed, in contrast with the single IR and Raman active extra band appearing in our spectra in the H_2 stretching region.

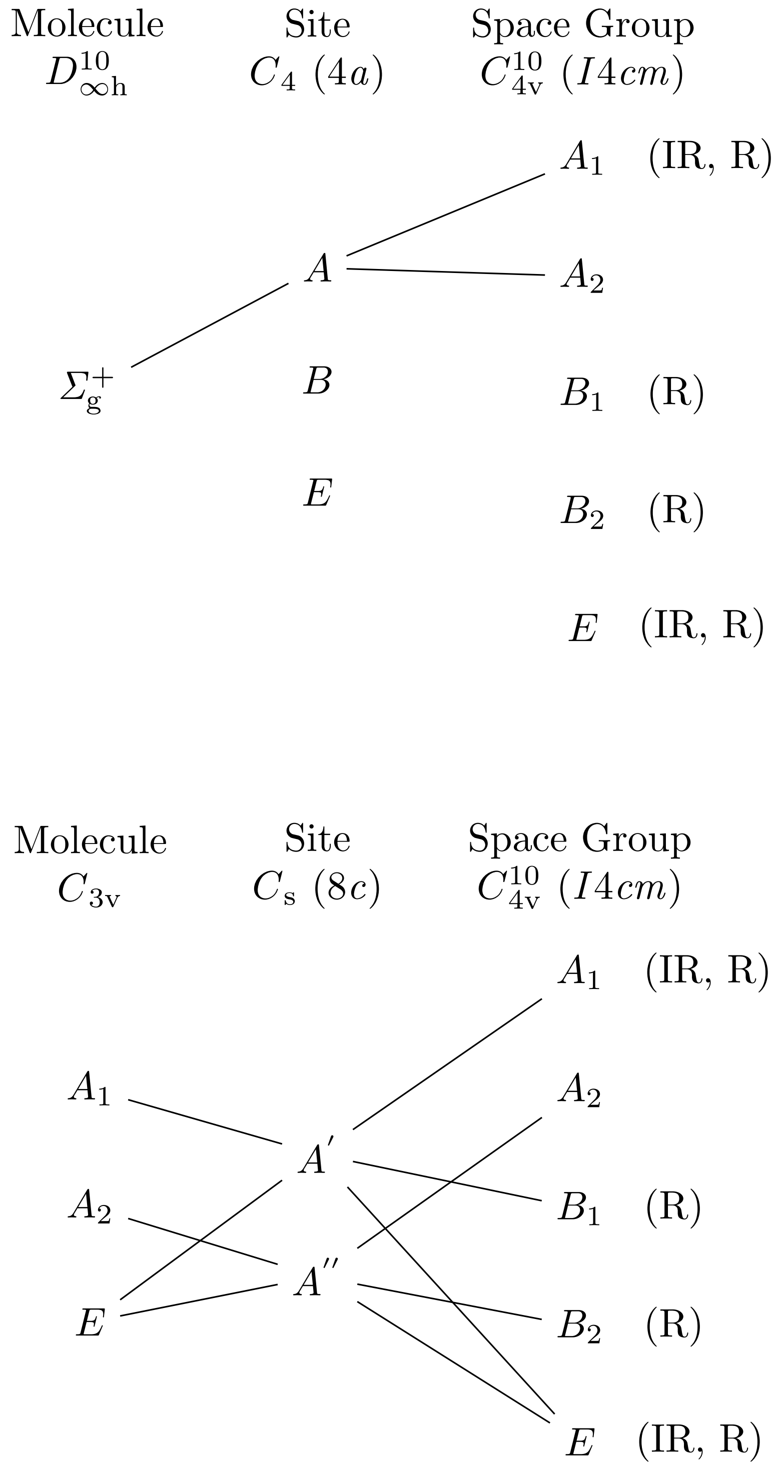

Supplementary Figure 10: Correlation diagrams between the irreducible representations of the normal modes of vibrations of H_2 (upper panel) and PH_3 (lower panel) in the point symmetry group of the isolated molecule (left side) and in the $I4cm$ (C_{4v}^{10}) space group of the $(PH_3)_2H_2$ crystal structure (right side), through the corresponding site symmetry and Wyckoff position (center). The infrared (IR) and Raman (R) optical activity of the symmetry species are also reported. One IR and Raman active component is expected for the H-H stretching vibration, as indeed observed in our IR and Raman spectra in the H_2 stretching region, thus supporting the presence of H_2 molecules on C_4 sites.

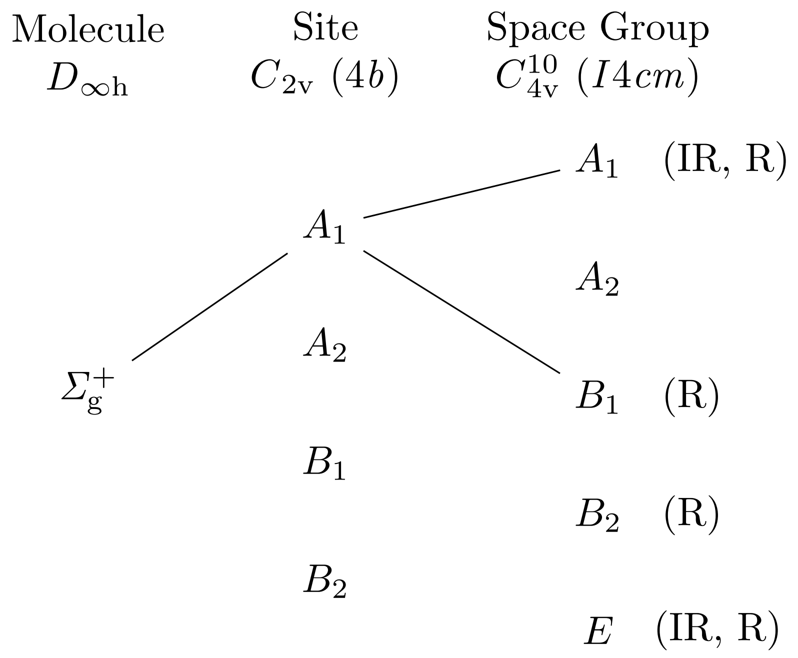

Supplementary Figure 11: Correlation diagram between the irreducible representations of the normal modes of vibrations of H_2 in the point symmetry group ($D_{\infty h}$) of the isolated molecule (left side) and in the $I4cm$ (C_{4v}^{10}) space group of the $(PH_3)_2H_2$ crystal structure (right side), through the hypothetical site with symmetry C_{2v} corresponding to 4b Wyckoff positions (center). The infrared (IR) and Raman (R) optical activity of the symmetry species are also reported. In this case, one IR and Raman active component (A_1) and one Raman active component (B_1), should be observed. The absence of the last one in our Raman spectra, supports the presence of H_2 molecules on the C_4 sites rather than on the C_{2v} ones.

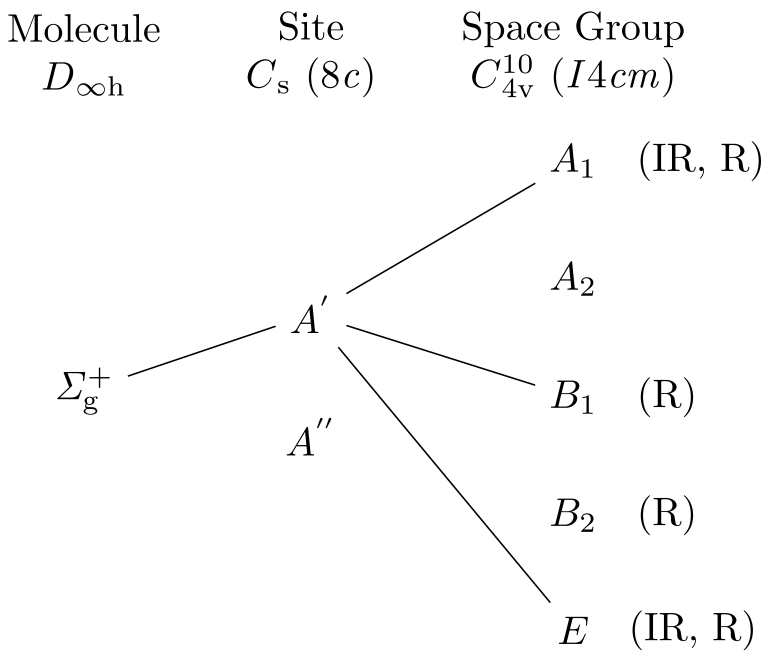

Supplementary Figure 12: Correlation diagram between the irreducible representations of the normal modes of vibrations of H_2 in the point symmetry group ($D_{\infty h}$) of the isolated molecule (left side) and in the $I4cm$ (C_{4v}^{10}) space group of the $(PH_3)_2H_2$ crystal structure (right side), through the hypothetical site with symmetry C_s corresponding to $8c$ Wyckoff positions (center). The infrared (IR) and Raman (R) optical activity of the symmetry species are also reported. In this case two IR and Raman active components (1 A_1, 1 E) and one Raman active components (1 B_1) should be observed, in contrast with the single IR and Raman active extra band appearing in our spectra in the H_2 stretching region, thus excluding the possibility of the H_2 molecules being located on the C_s sites.

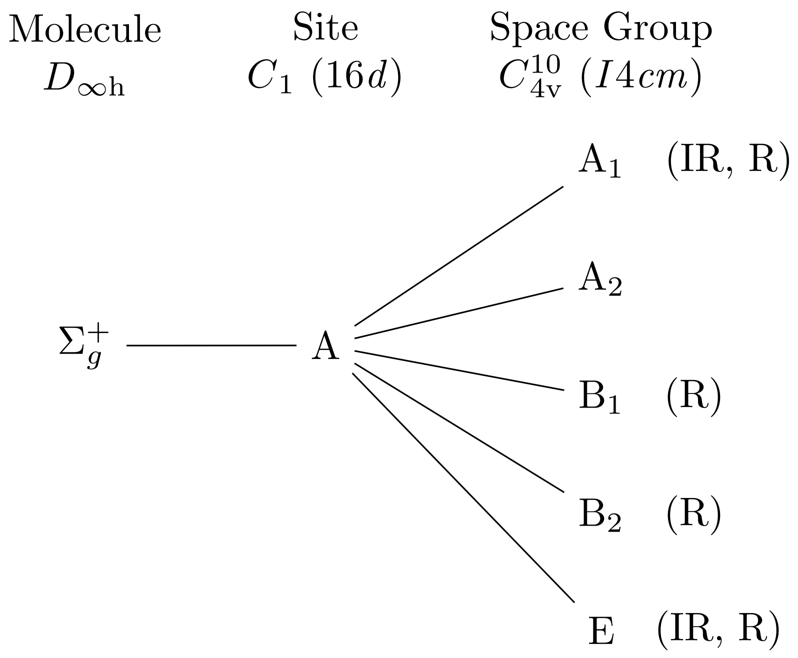

Supplementary Figure 13: Correlation diagram between the irreducible representations of the normal modes of vibrations of H_2 in the point symmetry group ($D_{\infty h}$) of the isolated molecule (left side) and in the $I4cm$ (C_{4v}^{10}) space group of the $(PH_3)_2H_2$ crystal structure (right side), through the hypothetical site with symmetry C_1 corresponding to 16d Wyckoff positions (center). The infrared (IR) and Raman (R) optical activity of the symmetry species are also reported. In this case three IR and Raman active components (1 A_1, 2 E) and two Raman active components (1 B_1 and 1 B_2) should be observed, in contrast to the single IR and Raman active extra band appearing in our spectra in the H_2 stretching region, thus excluding the possibility of the H_2 molecules being located on the C_1 sites.

Supplementary Note 7

Equation of state of $(PH_3)_2H_2$. Three XRD diffraction patterns of $(PH_3)_2H_2$ were collected, respectively at 4.1, 4.5 and 5.5 GPa after the crystallization of the reaction product. The structural data obtained from their refinement are reported in Supplementary Table 4.

The density values, calculated from the refined unit cell volume obtained using JANA 2006 software for Le Bail fit of the panoramic XRD pattern,[4] are: 1.166 g cm^{-3} at 4.1 GPa, 1.220 g cm^{-3} at 4.5 GPa and 1.271 g cm^{-3} at 5.5 GPa.

Supplementary Table 4: Unit cell parameters and volume of $(PH_3)_2H_2$ at at different pressures.

P (GPa)	a (Å)	b (Å)	c (Å)	α (degree)	β (degree)	γ (degree)	Volume (Å^3)
4.1	7.76788(16)	7.76788(16)	6.4584(2)	90	90	90	389.698(12)
4.5	7.70793(18)	7.70793(18)	6.4154(2)	90	90	90	381.137(9)
5.5	7.60348(8)	7.60348(8)	6.3305(1)	90	90	90	365.983(5)

The equation of state of $(PH_3)_2H_2$ in the 4.1-5.5 GPa pressure range, obtained by fitting of the pressure data point as a function of the volume using a 2nd order Birch-Murnaghan relation[19]

$$p(V) = \frac{3B}{2}\left[\left(\frac{V_0}{V}\right)^{\frac{7}{2}} - \left(\frac{V_0}{V}\right)^{\frac{5}{2}}\right] \tag{1}$$

indicates a bulk modulus B=6.7(0.8) GPa and volume V_0=536(14) Å^3 (Supplementary Figure 14).

The isothermal bulk modulus of $(PH_3)_2H_2$ is therefore comparable to that of other Al_2Cu alloy-type p-block element hydride-hydrogen phases (e.g. for $(H_2S)_2H_2$ it is 7.2 GPa[20]), but an order of magnitude higher than the bulk modulus of the phase I of H_2.[21]

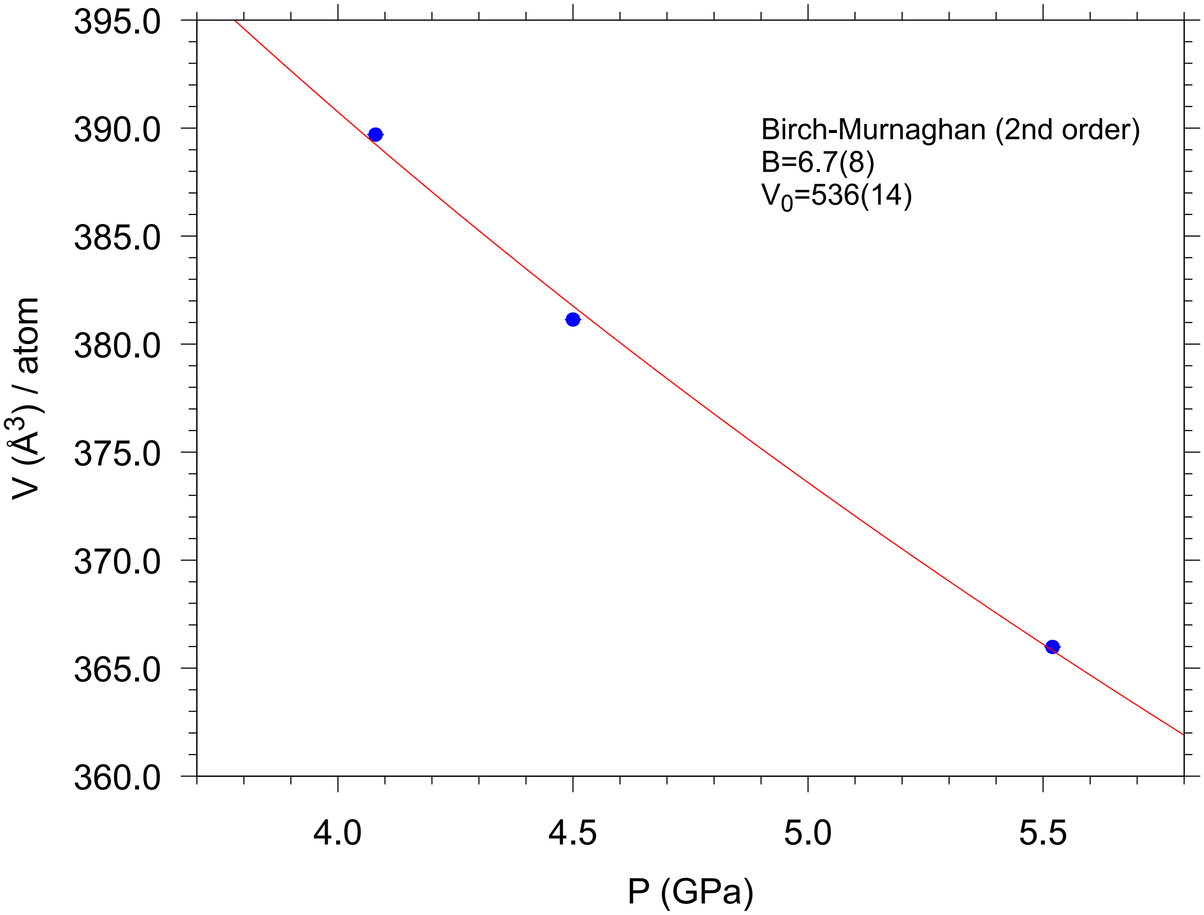

Supplementary Figure 14: Volume data points of $(PH_3)_2H_2$ at 4.1, 4.5 and 5.5 GPa (blue circles) and corresponding equation of state in the 4-6 GPa pressure range obtained by their fit using a 2nd order Birch-Murnaghan relation, with B=6.7±0.8 GPa and V_0=536±14 Å^3. The errors on the volume data points are of comparable size with the diameter of the blue solid circles.

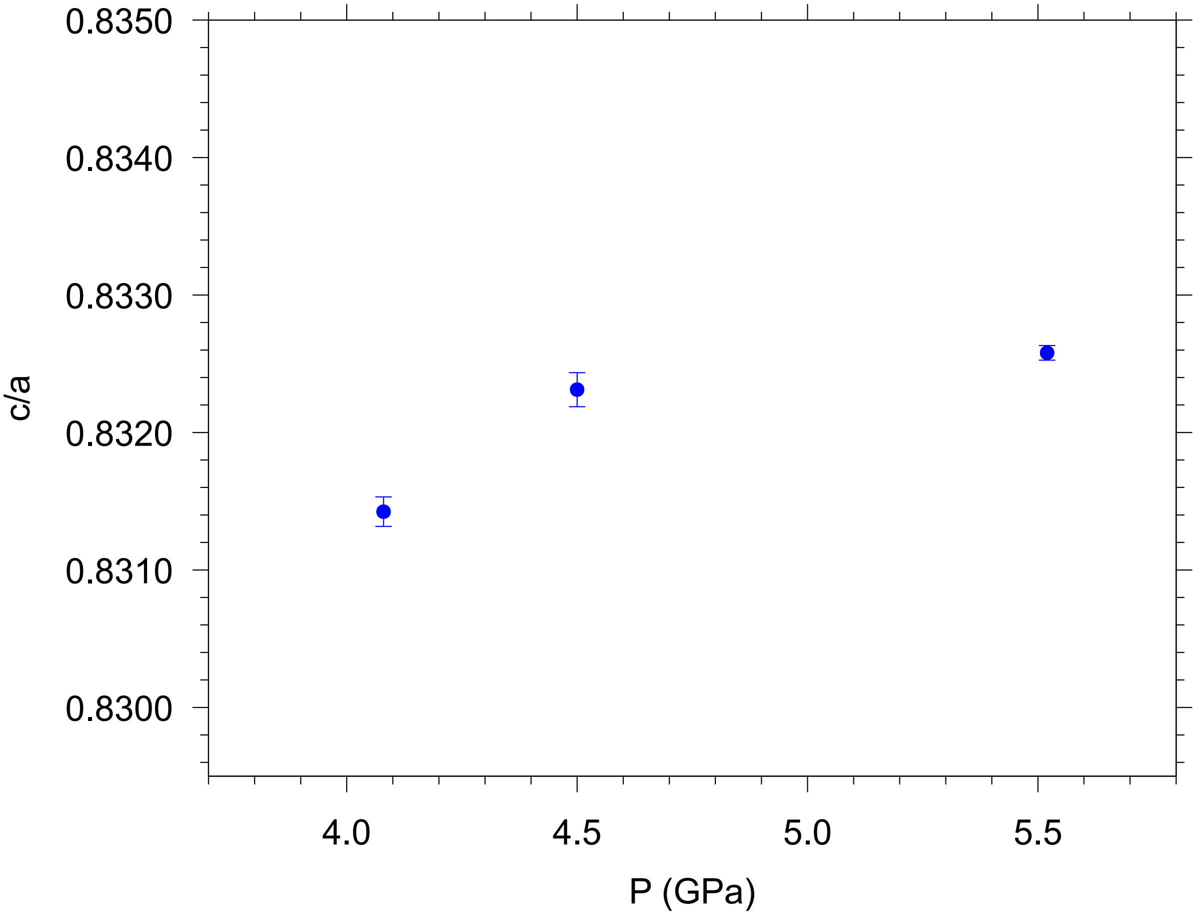

Supplementary Figure 15: Pressure evolution of the c/a axial ratio, indicating the almost isotropic compression within the applied pressure range.

The obtained lattice parameters allowed to conclude that the variation with pressure of the c/a ratio is nearly constant, indicating an almost isotropic compression within the applied pressure range (Supplementary Figure 15).

Supplementary Note 8

H_2-containing compounds of non-metallic elements.

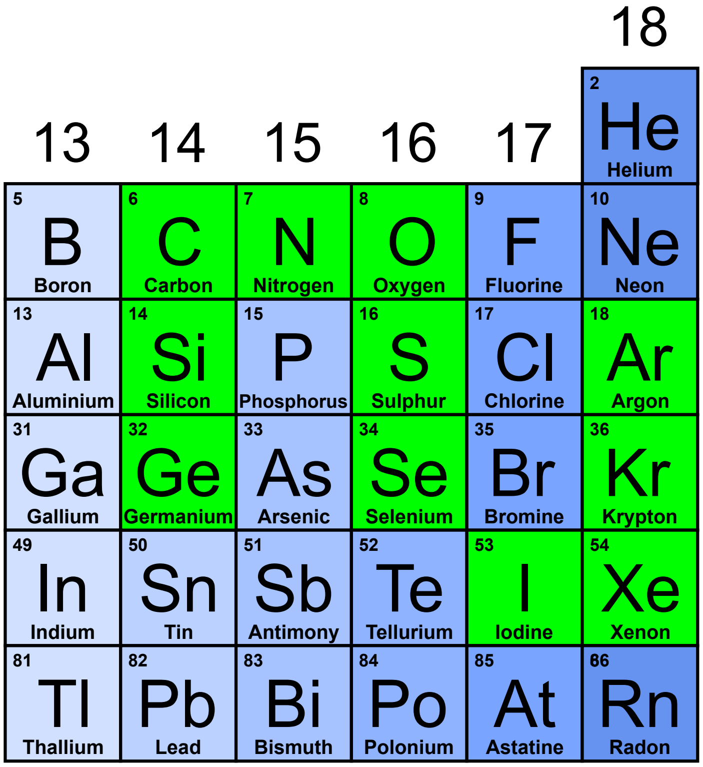

Supplementary Figure 16: Section of the Periodic Table of the Elements showing the experimentally reported non-metal elements able to form H_2-containing van der Waals hydrides, including noble gases, simple diatomics and molecular hydrides (green): $CH_4(H_2)_4$, CH_4H_2, $(CH_4)_2H_2$,[22] $SiH_4(H_2)_2$,[23] $GeH_4(H_2)_2$,[24] $N_2(H_2)_2$,[25] $(N_2)_6(H_2)_7$,[25,26] $(O_2)_3(H_2)_4$,[27] $(H_2O)_6H_2$,[28] $(H_2O)H_2$,[28] $(H_2S)_2H_2$,[20] $(H_2Se)_2H_2$,[29] $(HI)_2H_2$,[30] $Ar(H_2)_2$,[31] $Kr(H_2)_2$,[32] $Xe(H_2)_8$.[33,34]

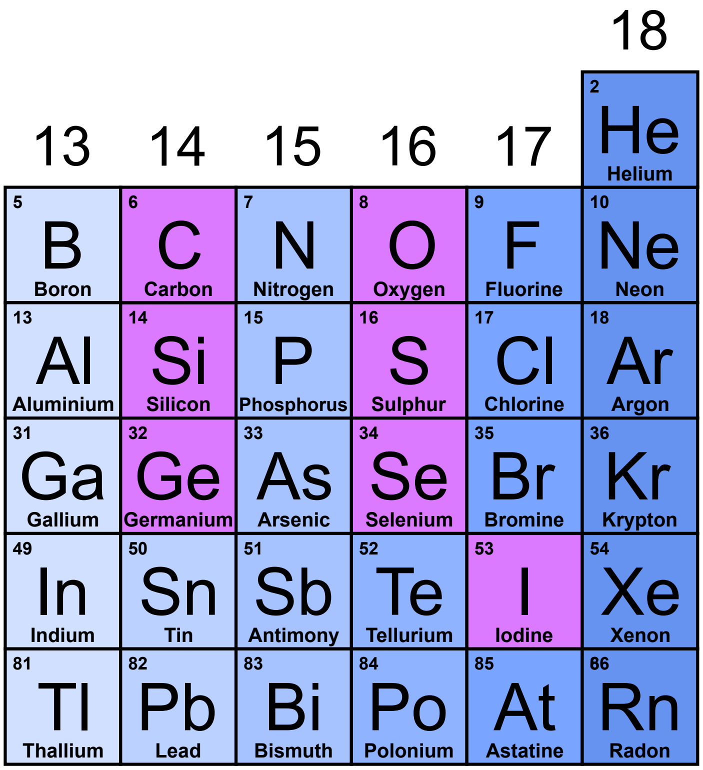

Supplementary Figure 17: Section of the Periodic Table of the Elements showing the experimentally reported non-metal elements able to form H_2-containing van der Waals compounds of the corresponding molecular hydrides (purple): $CH_4(H_2)_4$, CH_4H_2, $(CH_4)_2H_2$,[22] $SiH_4(H_2)_2$,[23] $GeH_4(H_2)_2$,[24] $(H_2O)_6H_2$,[28] $(H_2O)H_2$,[28] $(H_2S)_2H_2$,[20] $(H_2Se)_2H_2$,[29] $(HI)_2H_2$.[30]

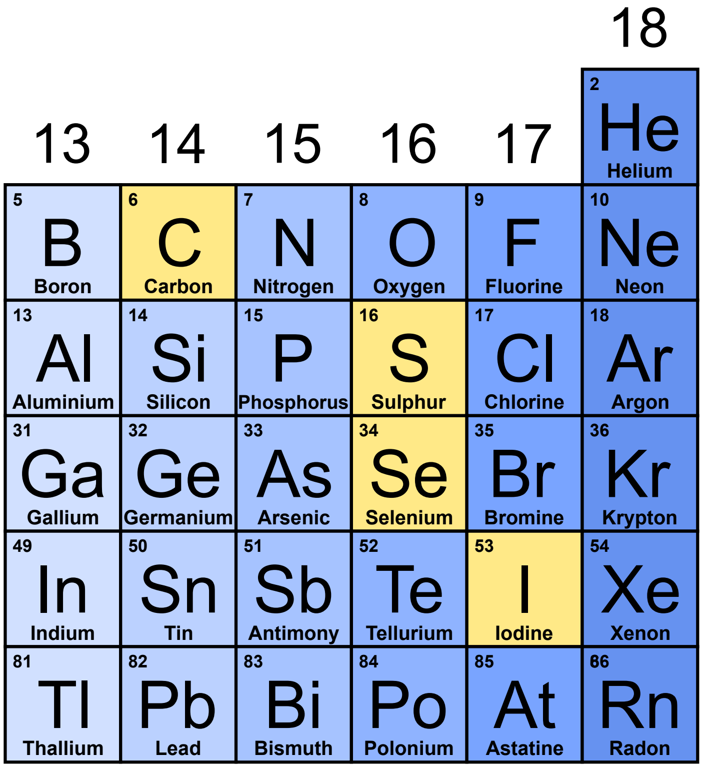

Supplementary Figure 18: Section of the Periodic Table of the Elements showing the experimentally reported non-metal elements able to form H_2-containing van der Waals compounds of the corresponding molecular hydrides with X_2H_2 composition (yellow) and $I4cm$ ($I4/mcm$) structure: $(CH_4)_2H_2$,[22] $(H_2S)_2H_2$,[20] $(H_2Se)_2H_2$,[29] $(HI)_2H_2$.[30]

Supplementary Note 9

The Haber-Bosch - like reaction for PH_3. As in the case of the Haber-Bosch process for the synthesis of NH_3 from N_2 and H_2,[35,36]

$$\frac{1}{2}N_2 + \frac{3}{2}H_2 \xrightarrow[\text{Fe catalyst}]{\text{450 °C, 100 atm}} NH_3 \tag{2}$$

the formation of PH_3 from P_{black} and H_2 reported in this study,

$$P_{black} + \frac{3}{2}H_2 \xrightarrow[\text{no catalyst}]{\text{1.2 GPa, 1000 K}} PH_3 \tag{3}$$

occurs under high pressure and high temperature conditions. In both cases, according to the Le Chatelier's principle, pressure shifts the reaction towards the formation of the reaction products with a reduced number of moles, while temperature likely allows to overcome kinetic barriers. Indeed no reactivity between P_{black} and H_2 was observed at high pressure before laser heating during the experimental time window. Within this pictures the formation of PH_3 from P and H_2 represents the analogue hydrogenation reaction of N_2, involving in this case the stable allotrope of the heavier pnictogen P, thus establishing a connection between the high pressure reactivity of the two group 15 elements and once again demonstrating the ability of pressure in enhancing the similarities in the properties of elements belonging to the same group and apparently exhibiting different behavior at ambient pressure. Going beyond this assertion is honestly not possible for several reasons, essentially related to the lack of thermochemical data about PH_3 and P_{black} at high pressure. Nevertheless, some important differences, worth to be noted, apply.

First, at standard conditions (0.1 MPa and 298.15 K), whereas the formation of NH_3 from N_2 and H_2 is exothermic ($\Delta_f H^\circ_{NH_3(g)}$= -46.11 kJ mol^{-1}) and exoergonic ($\Delta_f G^\circ_{NH_3(g)}$=

-16.45 kJ mol^{-1}), and thus thermodynamically favorable, the formation of gaseous PH_3 from solid P and H_2 is endothermic and endoergonic, thus thermodynamically unfavorable, either when starting from P_{white} ($\Delta_f H^\circ{}_{PH_3(g)}$=5.49 kJ mol^{-1}, $\Delta_f G^\circ{}_{PH_3(g)}$=13.4 kJ mol^{-1}), which is made of molecular P_4 units, or, even more, when starting from P_{black} ($\Delta_f H^\circ{}_{PH_3(g)}$=18.341 kJ mol^{-1}, $\Delta_f G^\circ{}_{PH_3(g)}$=20.8517 kJ mol^{-1}), which has a crystalline layered structure, made by the stacking of phopshorene layers with strong intra-layer covalent bonds (Supplementary Table 5). Considering the experimental observation of PH_3 from the high pressure and high temperature reaction of P_{black} and H_2 reported in this study, the positive value of the Gibbs energy at standard conditions suggests an even more important role of pressure in the formation of PH_3 than in the formation of NH_3.

Second, the Haber-Bosch process is a gas phase reaction, and all the thermodynamic considerations about enthalpy, entropy and Gibbs energy rely on this state of matter, whereas the reaction leading to the formation of PH_3 observed in our study occurs in condensed phase. As $\Delta_f H^\circ_{NH_3}$= $\Delta_r H^\circ_{NH_3}$= -46.11 kJ mol^{-1} and $\Delta_r S^\circ_{NH_3} = S^\circ_{NH_3} - \frac{1}{2}S^\circ_{N_2} - \frac{3}{2}S^\circ_{H_2}$= -99.4 J mol^{-1}, the formation of NH_3 from N_2 and H_2 remains spontaneous up to 463.9 K, when $\Delta_r G^\circ_{NH_3}$= $\Delta_r H^\circ_{NH_3}$ - T$\Delta_r S^\circ_{NH_3}$= 0. Nevertheless, even if the reaction is thermodynamically spontaneous at ambient conditions, the kinetic barrier to break the strong N_2 triple bond prevents the practical occurrence of the reaction. To overcome this issue, in the Haber-Bosch process high pressure is used for shifting the equilibrium towards the reactants according to

Supplementary Table 5: Thermodynamic data at 0.1 MPa and 298 K taken from[36] (* from ref[37]). Note that, despite P_{black} being the thermodynamically stable allotrope of P, the reference state for P in current use is P_{white}.[37]

	$\Delta_f H^\circ$ (kJ mol^{-1})	$\Delta_f G^\circ$ (kJ mol^{-1})	S° (J K^{-1} mol^{-1})
H_2 (g)	0	0	130.684
N_2 (g)	0	0	191.61
NH_3 (g)	-46.11	-16.45	192.45
PH_3 (g)	+5.49	+13.4	210.243
P (s, white)	0	0	41.09
P (s, black)*	-12.851	-7.338	22.586

the Le Chatelier's principle, while high temperature and catalysts are used to overcome the kinetic barrier. The combination of pressure and catalysts allows to optimize the temperature to be at the same time sufficiently high for speeding up the reaction while not making it unfavorable.

Supplementary Table 6: Thermodynamic data available for liquid (l) H_2,[38] N_2[39] and NH_3[40] at 5000 bar and 300 K and for liquid PH_3 at standard conditions (0.1 MPa, 298.15 K).[41] No high pressure thermochemical data are available for solid (s) P_{white} and P_{black}. The value marked by * was calculated in this SI section.

	$\Delta_f H^\circ$ (kJ mol^{-1})	$\Delta_f G^\circ$ (kJ mol^{-1})	S° (J K^{-1} mol^{-1})
H_2[38] (l)	15.896	-1.962	59.53
N_2[39] (l)	18.0449	-	112.49
NH_3[40] (l)	-3.26005	-19.4075*	89.81
PH_3[41] (l)	-9.50	25.36	120.1
P_{white} (s)	-	-	-
P_{black} (s)	-	-	-

High pressure data up to 5000 bar are available for H_2, N_2 and NH_3, but not for PH_3, P_4 or P_{black} (Supplementary Table 6).

At 5000 bar (≈0.5 GPa) and 300 K NH_3, N_2 and H_2 are liquid and the Gibbs energy for the reaction

$$\frac{1}{2}\mathrm{N_2} + \frac{3}{2}\mathrm{H_2} \longrightarrow \mathrm{NH_3} \tag{4}$$

expressed as

$$\Delta_r G_{NH_3} = \Delta_f G_{NH_3} - \frac{1}{2}\Delta_f G_{N_2} - \frac{3}{2}\Delta_f G_{H_2} = \Delta_r H_{NH_3} - T\Delta_r S_{NH_3} \tag{5}$$

where

$$\Delta_r H_{NH_3} = \Delta_f H_{NH_3} - \frac{1}{2}\Delta_f H_{N_2} - \frac{3}{2}\Delta_f H_{H_2} \tag{6}$$

$$\Delta_r S = S_{NH_3} - \frac{1}{2}S_{N_2} - \frac{3}{2}S_{H_2} \tag{7}$$

decreases to -19.4 kJ mol^{-1}, with respect to the standard conditions value of -16.3 kJ mol^{-1}, indicating that the reaction becomes increasingly favorable at high pressure in the liquid phase. The detailed calculations are reported in the following thermochemical equations for standard conditions (0.1 MPa, 298.15 K):

$$\Delta_{\mathrm{r}} H^{\circ}_{NH_3} = \Delta_{\mathrm{f}} H^{\circ}_{NH_3} - \frac{1}{2}\Delta_{\mathrm{f}} H^{\circ}_{N_2} - \frac{3}{2}\Delta_{\mathrm{f}} H^{\circ}_{H_2} = -46.11 - \frac{1}{2}(0) - \frac{3}{2}(0) = -46.11 \text{ kJ mol}^{-1} \quad (8)$$

$$\Delta_{\mathrm{r}} S^{\circ}_{NH_3} = S^{\circ}_{NH_3} - \frac{1}{2} S^{\circ}_{N_2} - \frac{3}{2} S^{\circ}_{H_2} = 192.45 - \frac{1}{2}(191.61) - \frac{3}{2}(130.684) = -99.381 \text{ J K}^{-1} \text{ mol}^{-1} \quad (9)$$

$$\Delta_{\mathrm{r}} G^{\circ}_{NH_3} = \Delta_{\mathrm{f}} G^{\circ}_{NH_3} - \frac{1}{2}\Delta_{\mathrm{r}} G^{\circ}_{N_2} - \frac{3}{2}\Delta_{\mathrm{r}} G^{\circ}_{H_2} = \Delta_{\mathrm{r}} H^{\circ}_{NH_3} - T\Delta_{\mathrm{r}} S^{\circ}_{NH_3} = -46.11 - (300)(-99.381 \cdot 10^{-3}) = -16.3 \text{ kJ mol}^{-1} \quad (10)$$

and for high pressure (5000 bar and 300 K):

$$\Delta_{\mathrm{r}} H_{NH_3} = \Delta_{\mathrm{f}} H_{NH_3} - \frac{1}{2}\Delta_{\mathrm{f}} H_{N_2} - \frac{3}{2}\Delta_{\mathrm{f}} H_{H_2} = -3.26005 - \frac{1}{2}(18.0449) - \frac{3}{2}(15.896) = -36.1265 \text{ kJ mol}^{-1} \quad (11)$$

$$\Delta_{\mathrm{r}} S_{NH_3} = S_{NH_3} - \frac{1}{2} S_{N_2} - \frac{3}{2} S_{H_2} = 89.81 - \frac{1}{2}(112.49) - \frac{3}{2}(59.53) = -55.73 \text{ J K}^{-1} \text{ mol}^{-1} \quad (12)$$

$$\Delta_{\mathrm{r}} G_{NH_3} = \Delta_{\mathrm{f}} G_{NH_3} - \frac{1}{2}\Delta_{\mathrm{r}} G_{N_2} - \frac{3}{2}\Delta_{\mathrm{r}} G_{H_2} = \Delta_{\mathrm{r}} H_{NH_3} - T\Delta_{\mathrm{r}} S_{NH_3} = -36.1265 - (300)(-55.73 \cdot 10^{-3}) = -19.4 \text{ kJ mol}^{-1} \quad (13)$$

A similar occurrence is expected also for the analogous reaction involving P_{black} instead of N_2 and PH_3 instead of NH_3, which is reported in this study

$$P_{\mathrm{black}} + \frac{3}{2}\,H_2 \longrightarrow PH_3 \tag{14}$$

In this case the expression of the Gibbs energy of reaction is

$$\Delta_{\mathrm{r}}G_{PH_3} = \Delta_{\mathrm{f}}G_{PH_3} - \Delta_{\mathrm{f}}G_{P_{black}} - \frac{3}{2}\Delta_{\mathrm{f}}G_{PH_3} = \Delta_{\mathrm{r}}H_{PH_3} - T\Delta_{\mathrm{r}}S_{PH_3} \tag{15}$$

where

$$\Delta_{\mathrm{r}}H_{PH_3} = \Delta_{\mathrm{f}}H_{PH_3} - \Delta_{\mathrm{f}}H_{P_{black}} - \frac{3}{2}\Delta_{\mathrm{f}}H_{H_2} \tag{16}$$

$$\Delta_{\mathrm{r}}S = S_{PH_3} - S_{P_{black}} - \frac{3}{2}S_{H_2} \tag{17}$$

Nevertheless, the absence of high pressure thermochemical data about P_{black} and PH_3, does not allow a quantitative calculation. A very rough qualitative estimation can be attempted by assuming a similar enthalpy and entropy for P_{black} at standard (0.1 MPa, 300 K) and high pressure (5000 bar, 300 K) conditions ($\Delta_{\mathrm{f}}H_{P_{black}} \approx \Delta_{\mathrm{f}}H^{\circ}_{P_{black}}$ and $S_{P_{black}} \approx S^{\circ}_{P_{black}}$), basing on the consideration that the A17 *Cmce* crystalline structure of P_{black} remains the same across this pressure range,[42] and a similar enthalpy and entropy for liquid PH_3 at standard (0.1 MPa, 300 K) and high pressure (5000 bar, 300 K) conditions ($\Delta_{\mathrm{f}}H_{PH_3} \approx \Delta_{\mathrm{f}}H^{\circ}_{PH3(liq)}$ and $S_{PH_3} \approx S^{\circ}_{PH3(liq)}$), for evaluating the following termochemical equations at standard conditions (0.1 MPa, 298.15 K):

$$\Delta_{\mathrm{r}} H^{\circ}_{PH_3} = \Delta_{\mathrm{f}} H^{\circ}_{PH_3} - \Delta_{\mathrm{f}} H^{\circ}_{P_{black}} - \frac{3}{2}\Delta_{\mathrm{f}} H^{\circ}_{H_2} = 5.49 - (-12.851) - \frac{3}{2}(0) = 18.3 \text{ kJ mol}^{-1} \tag{18}$$

$$\Delta_{\mathrm{r}} S^{\circ}_{PH_3} = S^{\circ}_{PH_3} - S^{\circ}_{P_{black}} - \frac{3}{2} S^{\circ}_{H_2} = 210.243 - (22.586) - \frac{3}{2}(130.684) = -8.369 \text{ J K}^{-1} \text{ mol}^{-1} \tag{19}$$

$$\begin{aligned} \Delta_{\mathrm{r}} G^{\circ}_{PH_3} = \Delta_{\mathrm{f}} G^{\circ}_{PH_3} - \Delta_{\mathrm{r}} G^{\circ}_{P_{black}} - \frac{3}{2}\Delta_{\mathrm{r}} G^{\circ}_{H_2} = \Delta_{\mathrm{r}} H^{\circ}_{PH_3} - T\Delta_{\mathrm{r}} S^{\circ}_{PH_3} = \\ 18.3 - (300)(-8.369 \cdot 10^{-3}) = 20.8 \text{ kJ mol}^{-1} \end{aligned} \tag{20}$$

and at high pressure (5000 bar and 300 K):

$$\begin{aligned} \Delta_{\mathrm{r}} H_{PH_3} = \Delta_{\mathrm{f}} H_{PH_3} - \Delta_{\mathrm{f}} H_{P_{black}} - \frac{3}{2}\Delta_{\mathrm{f}} H_{H_2} \\ \approx \Delta_{\mathrm{f}} H^{\circ}_{PH_3(liq)} - \Delta_{\mathrm{f}} H^{\circ}_{P_{black}} - \frac{3}{2}\Delta_{\mathrm{f}} H_{H_2} = \\ -9.50 - (-12.851) - \frac{3}{2}(15.896) = -20.5 \text{ kJmol}^{-1} \end{aligned} \tag{21}$$

$$\begin{aligned} \Delta_{\mathrm{r}} S_{PH_3} = S_{PH_3} - S_{P_{black}} - \frac{3}{2} S_{H_2} \\ \approx S^{\circ}_{PH_3(liq)} - S^{\circ}_{P_{black}} - \frac{3}{2} S_{H_2} = \\ 120.1 - (22.586) - \frac{3}{2}(59.53) = 8.219 \text{ J K}^{-1} \text{ mol}^{-1} \end{aligned} \tag{22}$$

$$\begin{aligned} \Delta_{\mathrm{r}} G_{PH_3} = \Delta_{\mathrm{f}} G^{\circ}_{PH_3} - \Delta_{\mathrm{f}} G^{\circ}_{P_{black}} - \frac{3}{2}\Delta_{\mathrm{f}} G_{H_2} = \Delta_{\mathrm{r}} H_{PH_3} - T\Delta_{\mathrm{r}} S_{PH_3} = \\ -20.493 - (300)(8.219 \cdot 10^{-3}) = -23.0 \text{ kJ mol}^{-1} \end{aligned} \tag{23}$$

According to this qualitative estimation $\Delta_{\mathrm{r}} G_{PH_3}$ becomes negative at 5000 bar, highlighting

the role of pressure in favoring the formation of PH_3, as in the case of NH_3. Furthermore, in contrast to the reaction of formation of NH_3, which has a negative $\Delta_\mathrm{r}S_{NH_3}$ at high pressure, the positive $\Delta_\mathrm{r}S_{PH_3}$ for the reaction of formation of PH_3 would suggest a role of temperature in the thermodynamic vs kinetic stability of the formation of PH_3.

It must be noted that the enthalpy of P_black reported by Wagman et al.[41] ($\Delta_\mathrm{f}H^\circ_{P_{black}}$= -39.3 kJ mol^{-1}) significantly differs with respect to the more recent value provided by Chase et al.[37] ($\Delta_\mathrm{f}H^\circ_{P_{black}}$= -12.851 kJ mol^{-1}), which was used in the previous thermochemical equations. If adopting the $\Delta_\mathrm{f}H^\circ_{P_{black}}$ value reported by Wagman et al., the following thermochemical data are obtained at standard conditions (0.1 MPa, 298.15 K):

$$\Delta_\mathrm{r}H^\circ_{PH_3} = \Delta_\mathrm{f}H^\circ_{PH_3} - \Delta_\mathrm{f}H^\circ_{P_{black}} - \frac{3}{2}\Delta_\mathrm{f}H^\circ_{H_2} = 5.49 - (-39.3) - \frac{3}{2}(0) = 44.8 \text{ kJmol}^{-1} \quad (24)$$

$$\Delta_\mathrm{r}S^\circ_{PH_3} = S^\circ_{PH_3} - S^\circ_{P_{black}} - \frac{3}{2}S^\circ_{H_2} = 210.243 - (22.586) - \frac{3}{2}(130.684) = -8.369 \text{ J K}^{-1}\text{ mol}^{-1} \quad (25)$$

$$\begin{aligned}\Delta_\mathrm{r}G^\circ_{PH_3} = \Delta_\mathrm{f}G^\circ_{PH_3} - \Delta_\mathrm{r}G^\circ_{P_{black}} - \frac{3}{2}\Delta_\mathrm{r}G^\circ_{H_2} = \Delta_\mathrm{r}H^\circ_{PH_3} - T\Delta_\mathrm{r}S^\circ_{PH_3} = \\ 44.8 - (300)(-8.369\cdot 10^{-3}) = 47.3 \text{ kJ mol}^{-1}\end{aligned} \quad (26)$$

and at high pressure (5000 bar and 300 K):

$$\begin{aligned}\Delta_\mathrm{r}H_{PH_3} = \Delta_\mathrm{f}H_{PH_3} - \Delta_\mathrm{f}H_{P_{black}} - \frac{3}{2}\Delta_\mathrm{f}H_{H_2} \approx \Delta_\mathrm{f}H^\circ_{PH_3(liq)} - \Delta_\mathrm{f}H^\circ_{P_{black}} - \frac{3}{2}\Delta_\mathrm{f}H_{H_2} = \\ -9.50 - (-39.3) - \frac{3}{2}(15.896) = 5.96 \text{ kJ mol}^{-1}\end{aligned} \quad (27)$$

$$\Delta_r S_{PH_3} = S_{PH_3} - S_{P_{black}} - \frac{3}{2} S_{H_2} \approx S^\circ_{PH_3(liq)} - S^\circ_{P_{black}} - \frac{3}{2} S_{H_2} = \\ 120.1 - (22.586) - \frac{3}{2}(59.53) = 8.219 \text{ J K}^{-1} \text{ mol}^{-1} \tag{28}$$

$$\Delta_r G_{PH_3} = \Delta_f G^\circ_{PH_3} - \Delta_f G^\circ_{P_{black}} - \frac{3}{2} \Delta_f G_{H_2} = \Delta_r H_{PH_3} - T \Delta_r S_{PH_3} = \\ 5.96 - (300)(8.219 \cdot 10^{-3}) = 3.49 \text{ kJ mol}^{-1} \tag{29}$$

Even if not becoming negative, also in this case $\Delta_r G_{PH_3}$ decreases with increasing pressure, highlighting the key role of pressure in favoring the reaction.

Finally, without appropriate experimental data and calculations, it can not be excluded that, in presence of unfavorable thermodynamics with an equilibrium constant $<<1$, pressure can shift the equilibrium towards the formation of PH_3 and trap the system into a kinetically controlled reactivity, with temperature helping to overcome energetic barrier.

Supplementary Note 10

IR based evidence of $(H_2S)_2H_2$ phase I to have $I4cm$ rather than $I4/mcm$ structure. During the reviewing period, we realized that that Pace et al. just published a paper about the phase diagram of $(H_2S)_2H_2$ at higher pressure than previously reported in literature.[43] Whereas the paper does not pay much attention to the low pressure region, it shows the first infrared spectra of $(H_2S)_2H_2$ in the crystal phase I, whose structure was proposed to be $I4/mcm$ by Strobel et al.[20] The pressure evolution of the infrared active vibrational frequencies and the infrared spectrum of the crystalline phase I at 7.2 GPa, reported respectively in Fig. 2 and 3 of the new paper by Pace et al., clearly show the presence of an infrared active band at $\sim$4150 cm^{-1}, which, according to group theory analysis performed in our study, is consistent with the $I4cm$ structure, but not with the $I4/mcm$ one. This new evidence supports our suggestion of phase I of $(H_2S)_2H_2$ (and hence likely also of $(H_2Se)_2H_2$ and $(HI)_2H_2$, see text) to be isostructural with $(PH_3)_2H_2$ and to belong to the $I4cm$ lower symmetry group rather than to the $I4/mcm$ higher symmetry one, as indeed hypothesized by Strobel et al. themselves.[20]

Even more recently, while studying the first room-temperature superconductor in the C-S-H system, Snider et al. in the Methods section of their article, under the "Simulations of initial photochemical products at 4.0 GPa" heading, noted that the previously reported $I4/mcm$ space group symmetry of $(H_2S)_2H_2$ structure seems too high, particularly concerning the positions of the H atoms.[44] Furthermore, considering the presence of weak H-bonding between H_2S molecules, in the $I4/mcm$ symmetry the arrangement of the H_2S molecules would violate the Bernal–Fowler "ice rules". The authors thus hypothesized that the actual symmetry of this phase may be lower than previously believed.

Acknowledgement

Thanks are expressed to EC through the European Research Council (ERC) for funding the project PHOSFUN "Phosphorene functionalization: a new platform for advanced multifunctional materials" (Grant Agreement No. 670173) through an ERC Advanced Grant. This study was supported also by the Deep Carbon Observatory (DCO) initiative under the project *Physics and Chemistry of Carbon at Extreme Conditions*, by the project "GreenPhos - alta pressione", by the Italian Ministero dell'Istruzione, dell'Università e della Ricerca (MIUR), by Ente Cassa di Risparmio dhave beeni Firenze under the project Firenze Hydrolab2.0 and by Fondazione Cassa di Risparmio di Firenze under the project HP-PHOTOCHEM. The authors acknowledge the European Synchrotron Radiation Facility (ESRF) for provision of synchrotron radiation facilities and thank G. Garbarino, M. Mezouar and J. Jacobs for assistance in using beamline ID27. The authors would like to thank Prof. Roberto Righini for useful discussions.

Supplementary References

(1) Prescher, C.; Prakapenka, V. B. DIOPTAS: a program for reduction of two-dimensional X-ray diffraction data and data exploration. *High. Press. Res.* **2015**, *35*, 223–230.

(2) Diffraction, R. O. CrysAlisPro Software system. *CrysAlisPro* **2018**, *version 1.171.39.46*.

(3) Sheldrick, G. M. Crystal structure refinement with *SHELXL*. *Acta Crystallogr. C* **2015**, *71*, 3–8.

(4) Petříček, V.; Dušek, M.; Palatinus, L. Crystallographic Computing System JANA2006: General features. *Z. Krist.-Cryst. Mater.* **2014**, *229*, 345–352.

(5) Natta, G.; Casazza, E. La struttura dell'idrogeno fosforato (PH_3) e dell'idrogeno arsenicale (AsH_3). *Gazz. Chim. Ital.* **1930**, *60*, 851 – 859.

(6) Ninet, S.; Datchi, F. High pressure–high temperature phase diagram of ammonia. *J. Chem. Phys.* **2008**, *128*, 154508.

(7) Cotton, F. A.; Wilkinson, G.; Murillo, C. A.; Bochmann, M. *Advanced inorganic chemistry*, 6th ed.; John Wiley & Sons, 1999.

(8) Chambers, C.; Holliday, A. K. *Modern inorganic chemistry*; Butterworth Scientific, 1982.

(9) Atkins, P.; Overton, T.; Rourke, J.; Weller, M.; Armstrong, F. *Shriver and Atkins' Inorganic Chemistry*, 5th ed.; W. H. Freeman, 2010.

(10) Weaver, J. R.; Parry, R. W. Dipole Moment Studies. IV. Trends in Dipole Moments. *Inorg. Chem.* **1966**, *5*, 718–723.

(11) Burrus, C. A. Stark Effect from 1.1 to 2.6 Millimeters Wavelength : PH3, PD3, DI, and CO. *J. Chem. Phys.* **1958**, *28*, 427–429.

(12) Wojdyr, M. *Fityk*: a general-purpose peak fitting program. *J. Appl. Cryst.* **2010**, *43*, 1126–1128.

(13) Winchester, R.; Whitby, M.; Shaffer, M. Synthesis of Pure Phosphorus Nanostructures. *Angew. Chem. Int. Ed.* **2009**, *48*, 3616–3621.

(14) Olego, D.; Baumann, J.; Kuck, M.; Schachter, R.; Michel, C.; Raccah, P. The microscopic structure of bulk amorphous red phosphorus: A Raman scattering investigation. *Solid State Commun.* **1984**, *52*, 311 – 314.

(15) Zaug, J. M.; Soper, A. K.; Clark, S. M. Pressure-dependent structures of amorphous red phosphorus and the origin of the first sharp diffraction peaks. *Nat. Mater.* **2008**, *7*, 890–899.

(16) Yuan, Y.; Li, Y.; Fang, G.; Liu, G.; Pei, C.; Li, X.; Zheng, H.; Yang, K.; Wang, L. Stoichiometric evolutions of PH_3 under high pressure: implication for high-T_c superconducting hydrides. *Natl. Sci. Rev.* **2019**, *6*, 524–531.

(17) Hahn, T. In *International Tables for Crystallography Volume A: Space Group Symmetry*, 5th ed.; Hahn, T., Ed.; Springer Netherlands: Dordrecht, 2005; Vol. A.

(18) Decius, J.; Hexter, R. *Molecular Vibrations in Crystals*; McGraw-Hill, 1977.

(19) Cliffe, M. J.; Goodwin, A. L. *PASCal*: a principal axis strain calculator for thermal expansion and compressibility determination. *J. Appl. Cryst.* **2012**, *45*, 1321–1329.

(20) Strobel, T. A.; Ganesh, P.; Somayazulu, M.; Kent, P. R. C.; Hemley, R. J. Novel Cooperative Interactions and Structural Ordering in H_2S-H_2. *Phys. Rev. Lett.* **2011**, *107*, 255503.

(21) Hemley, R. J.; Mao, H. K.; Finger, L. W.; Jephcoat, A. P.; Hazen, R. M.; Zha, C. S. Equation of state of solid hydrogen and deuterium from single-crystal x-ray diffraction to 26.5 GPa. *Phys. Rev. B* **1990**, *42*, 6458–6470.

(22) Somayazulu, M. S.; Finger, L. W.; Hemley, R. J.; Mao, H. K. High-Pressure Compounds in Methane-Hydrogen Mixtures. *Science* **1996**, *271*, 1400–1402.

(23) Strobel, T. A.; Somayazulu, M.; Hemley, R. J. Novel Pressure-Induced Interactions in Silane-Hydrogen. *Phys. Rev. Lett.* **2009**, *103*, 065701.

(24) Strobel, T. A.; Chen, X.-J.; Somayazulu, M.; Hemley, R. J. Vibrational dynamics, intermolecular interactions, and compound formation in GeH_4-H_2 under pressure. *J. Chem. Phys.* **2010**, *133*, 164512.

(25) Laniel, D.; Svitlyk, V.; Weck, G.; Loubeyre, P. Pressure-induced chemical reactions in the $N_2(H_2)_2$ compound: from the N_2 and H_2 species to ammonia and back down into hydrazine. *Phys. Chem. Chem. Phys.* **2018**, *20*, 4050–4057.

(26) Ciezak, J. A.; Jenkins, T. A.; Hemley, R. J. Optical and Raman microspectroscopy of nitrogen and hydrogen mixtures at high pressure. *AIP Conf. Proc.* **2009**, *1195*, 1291–1294.

(27) Loubeyre, P.; LeToullec, R. Stability of O_2/H_2 mixtures at high pressure. *Nature* **1995**, *378*, 44–46.

(28) Vos, W. L.; Finger, L. W.; Hemley, R. J.; Mao, H.-k. Novel H_2-H_2O clathrates at high pressures. *Phys. Rev. Lett.* **1993**, *71*, 3150–3153.

(29) Pace, E. J.; Binns, J.; Peña Alvarez, M.; Dalladay-Simpson, P.; Gregoryanz, E.; Howie, R. T. Synthesis and stability of hydrogen selenide compounds at high pressure. *J. Chem. Phys.* **2017**, *147*, 184303.

(30) Binns, J.; Liu, X.-D.; Dalladay-Simpson, P.; Afonina, V.; Gregoryanz, E.; Howie, R. T. Synthesis and stability of hydrogen iodide at high pressures. *Phys. Rev. B* **2017**, *96*, 144105.

(31) Ulivi, L.; Bini, R.; Loubeyre, P.; LeToullec, R.; Jodl, H. J. Spectroscopic studies of the $Ar(H_2)_2$ compound crystal at high pressure and low temperatures. *Phys. Rev. B* **1999**, *60*, 6502–6512.

(32) Kleppe, A. K.; Amboage, M.; Jephcoat, A. P. New high-pressure van der Waals compound $Kr(H_2)_4$ discovered in the krypton-hydrogen binary system. *Sci. Rep.* **2014**, *4*, 4989.

(33) Somayazulu, M.; Dera, P.; Goncharov, A. F.; Gramsch, S. A.; Liermann, P.; Yang, W.; Liu, Z.; Mao, H.-k.; Hemley, R. J. Pressure-induced bonding and compound formation in xenon–hydrogen solids. *Nat. Chem.* **2010**, *2*, 50–53.

(34) Somayazulu, M.; Dera, P.; Smith, J.; Hemley, R. J. Structure and stability of solid $Xe(H_2)_n$. *J. Chem. Phys.* **2015**, *142*, 104503.

(35) Büchel, K. H.; Moretto, H.-H. M.; Werner, D. *Industrial inorganic chemistry*, 2nd ed.; Wiley-VCH, 2000.

(36) Atkins, P.; de Paula, J.; Keeler, J. *Atkins Physical Chemistry*, 11th ed.; Oxford University Press, 2018.

(37) Chase, M. W. J. NIST-JANAF Themochemical Tables, Fourth Edition. *J. Phys. Chem. Ref. Data* **1998**, *Monograph 9*, 1348.

(38) Hemmes, H.; Driessen, A.; Griessen, R. Thermodynamic properties of hydrogen at pressures up to 1 Mbar and temperatures between 100 and 1000K. *J. Phys. C Solid State Phys.* **1986**, *19*, 3571.

(39) Jacobsen, R. T.; Stewart, R. B. Thermodynamic Properties of Nitrogen Including Liquid and Vapor Phases from 63K to 2000K with Pressures to 10,000 Bar. *J. Phys. Chem. Ref. Data* **1973**, *2*, 757–922.

(40) Haar, L.; Gallagher, J. S. Thermodynamic properties of ammonia. *J. Phys. Chem. Ref. Data* **1978**, *7*, 635–792.

(41) Wagman, D. D.; Evans, W. H.; Parker, V. B.; Schumm, R. H.; Halow, I.; Balley, S. M.; Churney, K. L.; Nuttal, R. L. The NBS Tables of Chemical Thermodynamic Properties: Selected Values for Inorganic and C1 and C2 Organic Substances in SI Units. *J. Phys. Chem. Ref. Data* **1982**, *11*, 1–407.

(42) Scelta, D.; Baldassarre, A.; Serrano-Ruiz, M.; Dziubek, K.; Cairns, A. B.; Peruzzini, M.; Bini, R.; Ceppatelli, M. Interlayer Bond Formation in Black Phosphorus at High Pressure. *Angew. Chem. Int. Ed.* **2017**, *56*, 14135–14140.

(43) Pace, E. J.; Liu, X.-D.; Dalladay-Simpson, P.; Binns, J.; Peña Alvarez, M.; Attfield, J. P.; Howie, R. T.; Gregoryanz, E. Properties and phase diagram of $(H_2S)_2H_2$. *Phys. Rev. B* **2020**, *101*, 174511.

(44) Snider, E.; Dasenbrock-Gammon, N.; McBride, R.; Debessai, M.; Vindana, H.; Vencatasamy, K.; Lawler, K. V.; Salamat, A.; Dias, R. P. Room-temperature superconductivity in a carbonaceous sulfur hydride. *Nature* **2020**, *586*, 373–377.
